# Cell trafficking and regulation of osteoblastogenesis by extracellular vesicle associated bone morphogenetic protein 2

**DOI:** 10.1002/jev2.12155

**Published:** 2021-10-20

**Authors:** Saigopalakrishna S. Yerneni, Juraj Adamik, Lee E. Weiss, Phil G. Campbell

**Affiliations:** ^1^ Department of Biomedical Engineering Carnegie Mellon University Pittsburgh Pennsylvania USA; ^2^ Division of Hematology/Oncology, Department of Medicine UPMC Hillman Cancer Center Pittsburgh Pennsylvania USA; ^3^ The Robotics Institute Carnegie Mellon University Pittsburgh Pennsylvania USA; ^4^ The McGowan Institute for Regenerative Medicine University of Pittsburgh Pittsburgh Pennsylvania USA; ^5^ Engineering Research Accelerator, College of Engineering Carnegie Mellon University Pittsburgh Pennsylvania USA

**Keywords:** bioprinting, bone morphogenetic protein 2, drug delivery, exosome, extracellular vesicle, osteogenesis

## Abstract

Extracellular vesicles (EVs) are characterized by complex cargo composition and carry a wide array of signalling cargo, including growth factors (GFs). Beyond surface‐associated GFs, it is unclear if EV intralumenal growth factors are biologically active. Here, bone morphogenetic protein‐2 (BMP2), loaded directly into the lumen of EVs designated engineered BMP2‐EVs (eBMP2‐EVs), was comprehensively characterized including its regulation of osteoblastogenesis. eBMP2‐EVs and non‐EV ‘free’ BMP2 were observed to similarly regulate osteoblastogenesis. Furthermore, cell trafficking experiments suggest rapid BMP2 recycling and its extracellular release as ‘free’ BMP2 and natural occurring BMP2‐EVs (nBMP2‐EVs), with both being osteogenic. Interestingly, BMP2 occurs on the EV surface of nBMP2‐EVs and is susceptible to proteolysis, inhibition by noggin and complete dissociation from nBMP2‐EVs over 3 days. Whereas, within the eBMP2‐EVs, BMP2 is protected from proteolysis, inhibition by noggin and is retained in EV lumen at 100% for the first 24 h and ∼80% after 10 days. Similar to ‘free’ BMP2, bioprinted eBMP2‐EV microenvironments induced osteogenesis in vitro and in vivo in spatial registration to the printed patterns. Taken together, BMP2 signalling involves dynamic BMP2 cell trafficking in and out of the cell involving EVs, with distinct differences between these nBMP2‐EVs and eBMP2‐EVs attributable to the BMP2 cargo location with EVs. Lastly, eBMP2‐EVs appear to deliver BMP2 directly into the cytoplasm, initiating BMP2 signalling within the cell, bypassing its cell surface receptors.

## 1 INTRODUCTION

Extracellular vesicles (EVs), ranging in size from nanometres (< 50 nm) to microns (∼5 μm), are secreted by essentially all cells in the body. EVs are a constituent within the cell microenvironment, occurring both in extracellular body fluids as soluble ‘liquid‐phase’ EVs (Grigor'eva et al., 2016; Jia et al., 2014; Théry et al., 2018; Vlassov et al., 2012) and immobilized to extracellular matrices as ‘solid‐phase’ EVs (Genschmer et al., 2019; Huleihel et al., 2016; Yerneni et al., 2019). EVs play a significant role in intercellular communication throughout life, both in health and disease, by acting as delivery vehicles between cells, transporting intraluminal and surface cargo, including DNA, RNA, proteins, lipids, proteoglycans, metabolites and organelles between cells (Van Niel et al., 2018). Because EVs evolved to deliver cargo, they have gained increasing attention in recent years as vehicles for delivering either native and/or engineered cargo components for therapeutic‐specific applications. (Ha et al., 2016) Encapsulation of both endogenous and exogenous therapeutic agents in the EV lumen protects them from inactivation within the extracellular environment via enzymatic degradation (Frokjaer & Otzen, 2005; Hung et al., 2019).

Within the complex cargo composition of EVs, numerous growth factors (GFs) have been identified as native EV cargo constituents. Bone morphogenetic proteins (BMPs) (Nahar et al., 2008), transforming growth factor beta (TGFβ) (D'angelo et al., 2001) and vascular endothelial growth factors (Nahar et al., 2008) have been identified as constituents in solid‐phase EVs immobilized in the extracellular matrix (ECM), while BMP2/4 (Draebing et al., 2018), TGFβ (Webber et al., 2010) Wnt proteins (Gross et al., 2012) hepatocyte growth factor (Thompson et al., 2013) and fibroblast growth factor (Javidi‐Sharifi et al., 2019) have been found in biological fluids as liquid‐phase EV constituents. Although such reports have established that GFs are associated with EVs, the use of EVs as GF delivery vehicles remains relatively unexplored. In regard to BMP2, Draebing *et al*. reported that the transport of BMP2/4 by EVs is an essential mechanism for developmental morphogenesis (Draebing et al., 2018). Wei *et al*. demonstrated that EVs secreted by BMP2‐treated macrophages exhibited dramatically improved osteogenic bioactivity in vitro, although they did not determine whether BMP2 was an EV cargo constituent (Wei et al., 2019). Huang *et*. *al*. genetically modified bone marrow‐derived mesenchymal stem cells to overexpress BMP2 and found that although secreted EVs did not contain BMP2, EVs were enriched in multiple osteogenic miRNAs (Huang et al., 2020). Others have engineered EVs to deliver BMP2 plasmid DNA (Liang et al., 2020). Naturally occurring EV surface‐presented BMP2 (Draebing et al., 2018) can directly interact with their corresponding receptors on the recipient cell membrane and presumably signalling intercellularly following endosomal trafficking, similar to EV surface bound TGFβ (Shelke et al., 2019). However, the potential pathway(s) mediating EV intraluminal GF signalling remain unclear. Understanding such signalling pathway(s) could potentially shed light on alternate mechanisms by which EV encapsulated intraluminal GFs control cell fate.

Beyond diffusible soluble EVs, ECM immobilized, solid‐phase EVs likely enable persistent tissue‐specific microenvironments that spatially localize their cell signalling cargo. Such microenvironments would therefore be critical for tissue maintenance and normal functioning, similar to how native (non‐EV‐associated) solid‐phase GFs in the ECM are involved in tissue homeostasis (Taipale & Keski‐Oja, 1997). Therefore, recapitulating not only native solid‐phase EV microenvironments but also those incorporating exogenous GF‐EVs would be a logical approach for biomimicking localized delivery of GF‐based therapeutics. Here, we report an extension of our bioprinting technology to create spatially defined solid‐phase EV‐based microenvironments (Yerneni et al., 2019; Yerneni et al., 2019) consisting of engineering EVs with intralumenally loaded exogenous BMP2 (eBMP2‐EVs).

Recombinant human BMP2 was selected as a paradigm GF due to its biological and clinical significance. The clinical application of BMP2 remains challenging, mainly because large pharmacological doses of BMP2 are required due to the poor binding of BMP2 to the collagen type I sponge, which results in burst release and short residence times in vivo (Uludag et al., 2001). This results in undesirable off‐target side‐effects, especially when BMP2 is used off‐label (James et al., 2016). The potential advantage of utilizing EV delivery vehicles for BMP2 are (i) the high binding capacity of EVs for ECM constituents, including collagen type I (Yerneni et al., 2019), and (ii) the ability of EVs to protect intraluminal cargo from extravesicular antagonists/inhibitor/enzyme degradation (Hung et al., 2019). Therefore, eBMP2‐EVs could potentially act as a BMP2 depot, requiring lower doses that might mitigate off‐target effects. Beyond the basic science aspects, this report lays the groundwork toward establishing and validating a methodology to engineer, deliver and study solid‐phase EVs containing intraluminal exogenous GFs. This work expands our previous studies using bioprinted spatially controlled solid‐phase non‐EV‐associated GF microenvironments which include extensive in vitro and in vivo application of bioprinted BMP2 as supported by representative publications (Cooper et al., 2010a; Miller et al., 2009; Phillippi et al., 2008; Tuzmen et al., 2018). We originally pioneered this biopatterning technology based on biomimicry to engineer microenvironments with protein and peptide‐based signalling molecules, and their response modifiers, to recapitulate aspects of biological spatial patterning of cell functions occurring during morphogenesis and tissue repair/regeneration. This approach has the potential to accelerate the application of EVs delivering GFs as practical therapeutics, particularly those based on localized solid‐phase delivery.

In this study, both liquid‐ and solid‐phase eBMP2‐EVs were studied. Loading efficiency, retention, and protection of exogenously loaded BMP2 in EVs was assessed using radiolabeling‐based assays. Radiolabeling, fluorescence, and gene/protein expression assays were performed to validate liquid‐phase eBMP2‐EV cell trafficking and osteogenic bioactivity in vitro. Moreover, the ability of eBMP2‐EVs to protect BMP2 from noggin, a natural BMP2 inhibitor (Walsh et al., 2010), was also assessed. Cell trafficking experiments using ‘free’ BMP2 (BMP2 not associated with EVs) confirmed that BMP2 incorporation into EV cargo occurs naturally, however, unlike eBMP2‐EVs, this naturally cell loaded form of BMP2‐EVs (nBMP2‐EVs), had the BMP2 unstably localized on the EV surface. Moreover, this EV surface presented BMP2 was inhibited by noggin. Bioprinting was used to create solid‐phase eBMP2‐EV microenvironments on collagen‐coated coverslips for in vitro studies and in collagen‐rich acellular dermal matrix (ADM) material for in vivo studies. In vitro, picogram level doses of intraluminal EV BMP2 in eBMP2‐EVs induced osteogenic differentiation in registration to bioprinted patterns. In vivo, nanogram level doses induced localized heterotopic ossification (HO) in a mouse muscle pocket model. Taking the in vitro experiments together, the data suggest that BMP2 cell signalling involves cell trafficking whereby cell internalized BMP2 is recycled as EV cargo, but that this nBMP2‐EVs exhibits distinct attributes compared to eBMP2‐EVs. The data further suggests a novel intracellular signalling mechanism for eBMP2‐EVs beyond the established GF signalling paradigm which is initiated by a GF binding to its cell surface receptor.

## 2 EXPERIMENTAL METHODS

Additional Materials and Methods are provided in the Supporting Information Section. These include EV cell culture source, isolation and characterization, as well as bioprinting and in vivo evaluations.

### 2.1 Iodination of BMP2

Recombinant human BMP2 (Medtronic, Inc., Minneapolis, MN) was iodinated via the chloramine T method (Miller et al., 2009) as adapted from the original iodination protocol established for TGFβ (Frolik et al., 1984). BMP2 (10 μg) was reacted with 500 μCi ^125^I‐Na at 25°C with stepwise addition of 3 aliquots of dilute chloramine T solution (100 μg/ml). The resulting ^125^I‐BMP2 was > 97 % trichloroacetic acid perceptible with minimal protein aggregate formation. Specific activity of ^125^I‐BMP2 was from 55–80 μCi/μg.

### 2.2 Loading of BMP2 into EVs

Two different physical loading methods, electroporation and sonication, were investigated for loading BMP2 into EVs (Haney et al., 2015; Tian et al., 2014). A mixture of 10 μg of EVs and 1 μg BMP2 were used for these experiments. For electroporation, the mixture was electroporated (Bio‐Rad Laboratories, Hercules, CA) at 1 kV for 5 ms, and then incubated at 37°C for 1 h to allow membrane recovery. For sonication, the mixture was sonicated (Tekmar sonic disruptor) on ice using a 0.25″ tip at 20% amplitude, 6 cycles of 30 s on/off for 3 min with a 2 min cooling period between each cycle. The unloaded BMP2 was removed using a 100,000 kDa MWCO membrane filter (Vivaspin^®^ columns, Sartorius AG, Göttingen, Germany). EV surface‐bound BMP2 was removed by pH 3.0 acid‐incubation followed by separation of EVs from BMP2 using mini‐SEC. Loading of BMP2 in EVs was confirmed by immunoblotting and ^125^I‐BMP2.

### 2.3 Retention of BMP2 in EVs

^125^I‐BMP2 was loaded into EVs using the procedures described above. Approximately 50 μl of ^125^I‐BMP2 corresponding to ∼1 × 10^7^ cpm was mixed with 1 μg of unlabelled BMP2. Immediate post‐loading and purification, aliquots of ^125^I‐BMP2/BMP2/EVs containing ∼100,000 cpm ^125^I‐BMP2 were aliquoted into 12 × 75 mm polypropylene tubes containing simulated body fluid (SBF; composition: 10 % FBS, 0.02 % sodium azide, 25 mM HEPES in DMEM) to a total 1 ml volume. Tubes were incubated at 37°C and samples were eluted using Sepharose 2B SEC with PBS at 23°C at different time points, with a primary focus on early time points but going out to 14 days.

Radioactivity associated with EV and protein fractions were determined. The percent ^125^I‐BMP2 retained in EVs was expressed as the percentage of radioactivity associated with the EV peak over the combined EV and protein peaks. The zero timepoint was determined immediately post purification and data was normalized to percent ^125^I‐BMP2 retained in EVs. To confirm intralumenal localization of ^125^I‐BMP2 in EVs, ^125^I‐eBMP2‐EVs were treated with 1 mM trypsin, for a minimum of 30 min, 37°C, then eluted over 2B Sepharose. The resulting radioactivity associated with the EV fraction was compared to the EV fraction of a non‐protease control group. The proteolytic sensitivity of ‘free’ ^125^I‐BMP2 was confirmed by treating ^125^I‐BMP2 with trypsin under similar conditions, then conducting TCA precipitation to access degradation. ^125^I‐eBMP2‐EVs were also treated with 0.1 % Triton‐X100 for a minimum of 15 min, 25 C, followed by SEC to confirm detergent‐based disruption of ^125^I‐eBMP2‐EVs.

### 2.4 Radiolabeled‐based cell binding and cell trafficking experiments

Confluent cultures of C2C12 cells in 6‐well plates were rinsed twice with PBS and incubated in 1 ml cell binding buffer (0.1 M HEPES 0.12 M NaCl, 5 mM KCl, 1.2 mM MgSO_4_, 8 mM glucose, 1% BSA, pH 7.4 (Campbell & Novak, 1991) under 37°C in CO_2_ cell culture conditions for 1 h. Buffer was aspirated and fresh binding buffer was added along with ∼100,000 cpm of ^125^I‐eBMP2‐EVs. At indicated time points, representative plates were placed on ice, buffer was aspirated and cells were rinsed three times with ice‐cold PBS. 1 ml ice‐cold acid wash buffer (50 mM glycine HCl, 01 M NaCl, pH 3) was added to each well and cells were incubated on ice for 10 min. Acid buffer was collected into 12 × 75 mm polypropylene tubes for radioactive determination. This represented the portion of ^125^I‐eBMP2‐EVs bound to the cell surface. NaOH (1 M) was added at 1 ml per well and cells incubated with agitation for 30 min at RT. Solubilized cells were then transferred to 12 × 75 mm polypropylene tubes for radioactive determination. This represented the portion of ^125^I‐eBMP2‐EVs internalized within cells. Data was presented as total cell associated, cell surface and cell internalized radioactivity.

For cell trafficking experiments, we were inspired by the protocols used to study epidermal growth factor cell trafficking (Pinilla‐Macua & Sorkin, 2015). ^125^I‐BMP2 or ^125^I‐eBMP2‐EVs were associated with cells as described above, excepting that once the acid rinse step was collected, cells containing internalized ^125^I‐BMP2 or ^125^I‐eBMP2‐EVs were transitioned from ice to 37 °C incubation with fresh cell binding buffer. After 30 min at 37 °C, the cells were again placed on ice and the buffer was collected for counting, which represents the recycled ^125^I‐BMP2,^125^I‐nBMP2‐EVs or ^125^I‐eBMP2‐EVs that is released from the cell intracellular compartment into the buffer. The retained cell layer was incubated with ice‐cold acid rinse buffer for 10 min, 4 °C. The acid wash was collected for counting, which represents the recycled ^125^I‐BMP2, ^125^I‐nBMP2‐EVs or ^125^I‐eBMP2‐EVs bound to the cell surface. This by convention is used to also represent the recycled BMP2 ‘receptors’. The remaining intracellular radioactivity was determined using 1 N NaOH. Recycling data was presented as either percent of internalized ^125^I‐BMP2, ^125^I‐nBMP2‐EVs or ^125^I‐eBMP2‐EVs recycled, and the percent of cell surface or released recycled radioactivity. Trichloroacetic acid (TCA) precipitation was performed on recycled buffer to assess degradation of cell trafficked ^125^I‐BMP2. Specifically regarding ^125^I‐BMP2, in some experiments the released recycled radioactivity was pooled after initial counting, concentrated using a 3 kDa spin filter, the passed over SEC to separate the EV and protein fractions. The radioactivity associated with the EV fraction was expressed as recycled ^125^I‐nBMP2‐EV. Aliquots of recycled ^125^I‐nBMP2‐EVs were accessed for their ability to bind and be internalized in C2C12 cells. Aliquots were also immunoprecipitated against anti‐CD81 to confirm the association of radioactivity with EVs. Additionally, unlabelled 100 ng BMP2 was incubated with C2C12 cells and 30 min recycled nBMP2‐EV and BMP2 protein fraction was derived. The osteogenic ability of recycled BMP2 was assessed using alkaline phosphatase assay.

### 2.5 Osteogenic differentiation assays

#### 2.5.1 Alkaline phosphatase (ALP) assay

C2C12 cells were incubated with indicated treatments, washed with PBS to remove culture medium, and fixed for 20 min with 10% neutral buffered formalin (Millipore‐Sigma). Alkaline phosphatase activity was detected using a leukocyte alkaline phosphatase assay kit according to the manufacturer's instructions (Millipore‐Sigma, St. Louis, MO). Where required, ALP‐stained images were converted to CMYK format since this colour format is representative of reflected light colours as opposed to emitted light colours (RGB). Since the combination of cyan and magenta form the colour blue, these channels were added together and inverted. The average pixel intensity was determined using the image histogram tool in Adobe^®^ Photoshop 7.0 (Adobe^®^ Systems, San Jose, CA).

#### 2.5.2 Mineralization assay

MC3T3‐E1 (subclone 4) cells were seeded in growth media (ascorbic acid‐free α‐MEM, 10% FBS, 1% PS). After 24 h post‐seeding, differentiating media (alpha‐MEM, 10% FBS, 50 μg/ml ascorbic acid, 10 mM β‐glycerophosphate, 1% PS) was added with indicated treatments. Media (supplemented with respective treatments) was changed every 72 h. On day 21, cells were fixed in 10 % neutral buffered formalin, washed with distilled water three times, and alizarin red stain (Millipore‐Sigma, St. Louis, MO) was added to the wells and incubated for 1 h at RT. After imaging the cells, quantification of mineralization was performed using an osteogenesis quantitation kit (Millipore‐Sigma, St. Louis, MO) according to manufacturer's instructions. Briefly, alizarin red stained cells were treated with 10% acetic acid solution for 30 min with shaking, cells were scraped, centrifuged and the dissolved alizarin stain was quantified by measuring the OD at 405 nm (TECAN plate reader, Männedorf, Switzerland) using alizarin red reference standards.

### 2.6 Real‐time quantitative PCR (qPCR)

RNA from MC3T3 and C2C12 cells was isolated using a RNeasy Mini Kit (Qiagen, Hilden, Germany) according to the manufacturer instructions. First‐Strand cDNA Synthesis System (Life Technologies, Carlsbad, CA) was used for cDNA synthesis. qPCR was carried out using 2x Maxima SYBR Green/ROX qPCR Master Mix (Thermo Fisher Scientific, Waltham, MA) in Fast 96‐Well Reaction Plates (Applied Biosystems, Foster City, CA) using a StepOnePlus (Applied Biosystems, Foster City, CA). Relative mRNA levels were calculated using the ΔΔCt method using 18srRNA for normalization. The qPCR primers are listed in Table S1.

### 2.7 Immunoblotting

Immunoblotting was performed according to previously published protocols (Adamik et al., 2017) using the following antibodies directed towards: rhBMP2 (Abcam, 214821), RUNX2 (CST, D1L7F), p‐SMAD1/5‐S463/465 (CST, 41D10), TSG101 (Abcam, 125011), CD63 (Abcam, ab216130), CD9 (Abcam, 192726), β‐ACTIN (Sigma, 85316), p38 (SC, sc‐535), p‐p38‐Thr180/Tyr182 (CST, 4511), ERK (Santa Cruz, sc‐94) and p‐ERK1/2‐Thr202/Tyr204 (CST, 4370). Band signals were detected using Amersham ECL Western Blotting Detection Reagent (GE Life Sciences, Marlborough, MA) and analysed and quantitated using ProteinSimple Imager and AlphaView software (ProteinSimple, San Jose, CA). Full western blot images are shown in Figures S1‐5.

### 2.8 In vivo assessment in a mouse muscle pocket model

C57BL/6 male mice (n = 5; 22–26 grams) were utilized for evaluation of ADM scaffolds bioprinted with eBMP2‐EVs or EVs alone implanted in a murine muscle pocket. Animal care and experimental procedures were carried out at Carnegie Mellon University (Pittsburgh, PA) in accordance with the NIH Guide for the Care and Use of Laboratory Animals under an approved Institutional Animal Care and Use Committee (IACUC) protocol. eBMP2‐EVs or native EVs were printed at a total concentration of 150 ng EVs (containing 5 ng BMP2) per 4.5 mm ADM disc. Post bioprinting, overnight rinsing was performed to wash‐off unbound EVs prior to the implantation. Surgeries on each mouse were performed on both hind legs, with one leg serving as a non‐printed control. The thigh areas were first shaved to remove hair then treated with antiseptic agent (Povidone iodine 10%, Clinipad Corporation, Guilford, CT) followed by 70% ethyl alcohol. The thigh muscle on the dorsal side (Biceps femoris) was surgically exposed and a pocket was made in that muscle using a pair of forceps. ADM alone or ADM bioprinted with eBMP2‐EVs was folded in half, inserted into the pocket and a single suture (Proline^®^ 6‐0) was used to close the opening of the pocket. The wound was closed with interrupted sutures. The treatments (eBMP2‐EVs or EV alone controls) were randomized in each leg. Following the surgery, animals were given 1 mg/ml paediatric ibuprofen (Rite Aid, Camp Hill, PA) for pain through their drinking water. Locomotion, grooming, and eating habits of the animals were monitored post‐surgery. Animals were euthanized at 4 weeks via CO_2_ inhalation. For tissue harvest, the skin was removed from the legs, which were separated from the mouse at the hip sockets and fixed in 10% buffered formalin for 4 days at RT prior to μCT analysis.

### 2.9 Statistics

Data are presented as the mean ± SEM (n is as indicated in figure legends). One‐way analysis of variance (ANOVA) was used for data analysis to determine any statistically significant differences between two and multiple groups with Tukey's post‐hoc analysis where appropriate using GraphPad Prism (v8.0) software. *P* ≤ 0.05 was considered significant.

## 3 RESULTS

### 3.1 EV isolation, loading and characterization

**FIGURE 1:**
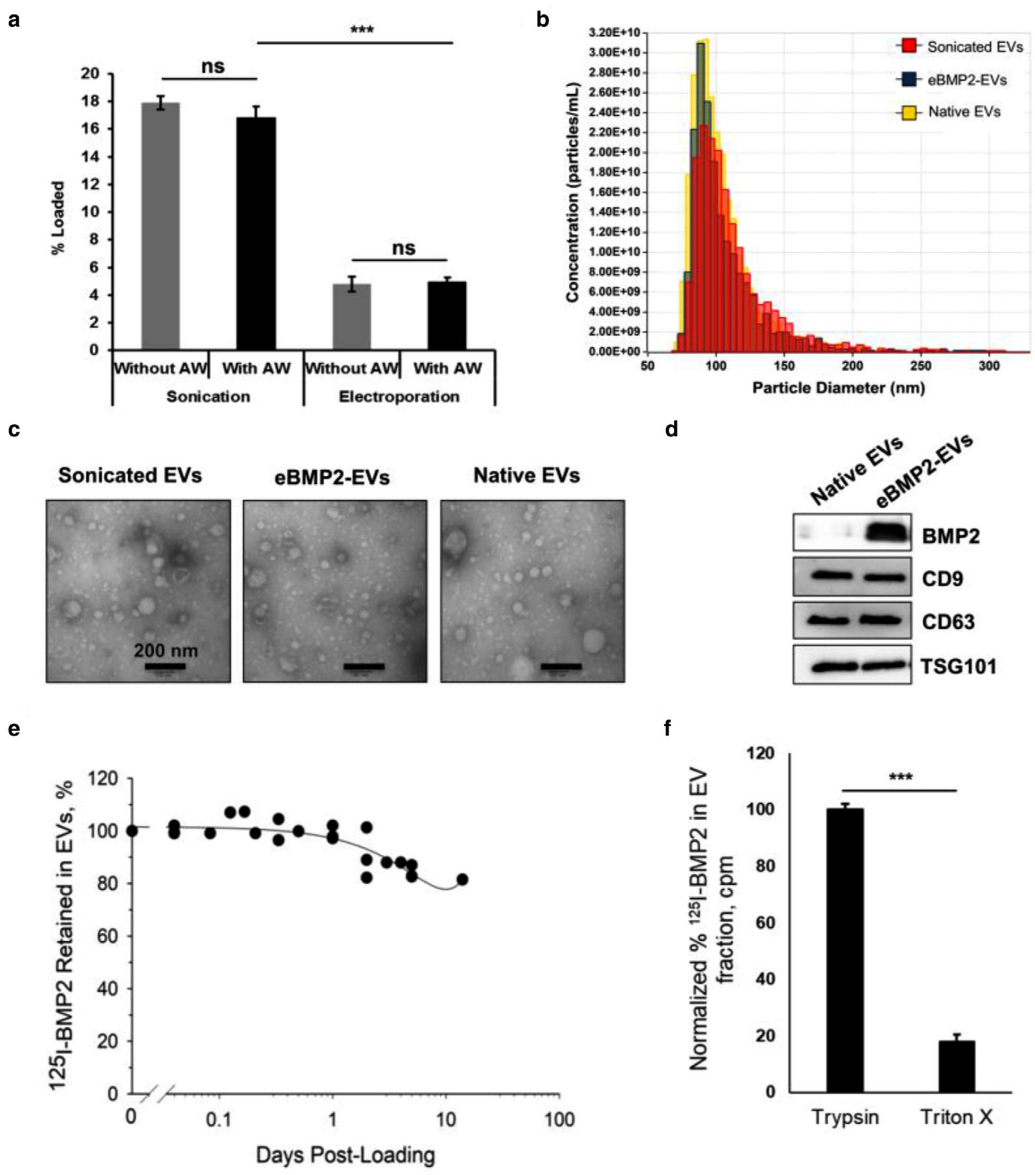
EV characterization and loading. (a) ^125^I‐BMP2 loading efficiency in EVs using sonication and electroporation techniques. Shown is a representative experiment for 3 independent biological experiments with error bars indicating SEM for three replicates. *** = *P* ≤ 0.001; ns = no significant difference; AW = acid washed. (b) Nanoparticle analysis of EVs pre‐ and post‐loading using tunable resistive pulse sensing. Sonicated EVs refers to EVs subjected to sonication in the absence of BMP2, eBMP2‐EVs refers to EVs sonicated in the presence of BMP2 and native EVs refer to non‐sonicated EVs. (c) Representative transmission electron microscopy images of EVs showing vesicular morphology. (d) Western blotting characterization of native EVs and eBMP2‐EVs. Images show immunoblots for presence of BMP2 and exosome markers CD9, CD63 and TSG101. (e) Retention of ^125^I‐BMP2 in EVs in simulated body conditions. Plot indicates the composite of 5 combined experiments. (f) Protection of ^125^I‐BMP2 encapsulated in EVs from trypsin and triton X‐100. Bars indicate mean ± SEM (5 independent experiments), *** = ρ≤0.001

The murine J774A.1 monocytic cell line, in its inactivated Mo state, served as our source of EVs. The average size of EVs, isolated from J774A.1 cells, was 100 nm and the EV protein concentration was 75 ‐ 100 μg/ml. BMP2 was loaded into EVs either by electroporation or sonication (Figure 1a). To evaluate the loading efficiency of BMP2 into EVs, ^125^I‐BMP2 was utilized. Extensive acid washing post‐loading was performed to release any external membrane‐bound BMP2, leaving only internalized intraluminal BMP2. BMP2 loading efficiencies were 18% ± 1.9% with sonication and 5% ± 0.7% with electroporation (Figure 1a). Since sonication resulted in over 3‐fold higher loading efficiency, sonication was utilized for BMP2‐EV formulation for all subsequent experiments. Tunable resistive pulse sensing (TRPS) analysis revealed that there was no drastic change in EV size distribution profile between pre‐ and post‐sonication (Figure 1b). Transmission electron microscopy (TEM) analysis demonstrated no difference between pre‐sonication native EVs and sonicated EVs with and without BMP2 (Figure 1c). Exosome markers TSG101, CD63 and CD9 were identified via western blot analysis and were similar between native EVs and eBMP2‐EVs (Figure 1d). Western blot analysis of BMP2 demonstrated the presence of BMP2 only in eBMP2‐EVs (Figures 1d and S1A‐D). The retention of BMP2 in eBMP2‐EVs was evaluated using ^125^I‐BMP2. Essentially 100% of BMP2 was retained within the EV lumen during the first 24 h of incubation in simulated body fluid (SBF) at 37°C with a gradual loss thereafter. Approximately 80% of the loaded BMP2 was retained in the EVs after 10 days post loading (Figure 1e). The loaded eBMP2‐EVs were also subjected to protease (trypsin) treatment followed by separation using size exclusion chromatography (SEC) to further confirm that the BMP2 in eBMP2‐EVs was intraluminal. We observed complete protection of EV‐encapsulated BMP2 from trypsin, whereas > 95% of ‘free’ BMP2 was degraded based on TCA precipitation (data omitted for simplicity). Detergent treatment with Triton‐X 100 further confirmed that BMP2 was contained within EVs. These data suggest that our loading protocol results in intraluminal loading of BMP2 in EVs and demonstrate successful non‐cell loading of BMP2 within cell secreted EVs, with minimal impact on measured EV properties.

### 3.2 eBMP2‐EVs are internalized by C2C12 and MC3T3 cells

**FIGURE 2:**
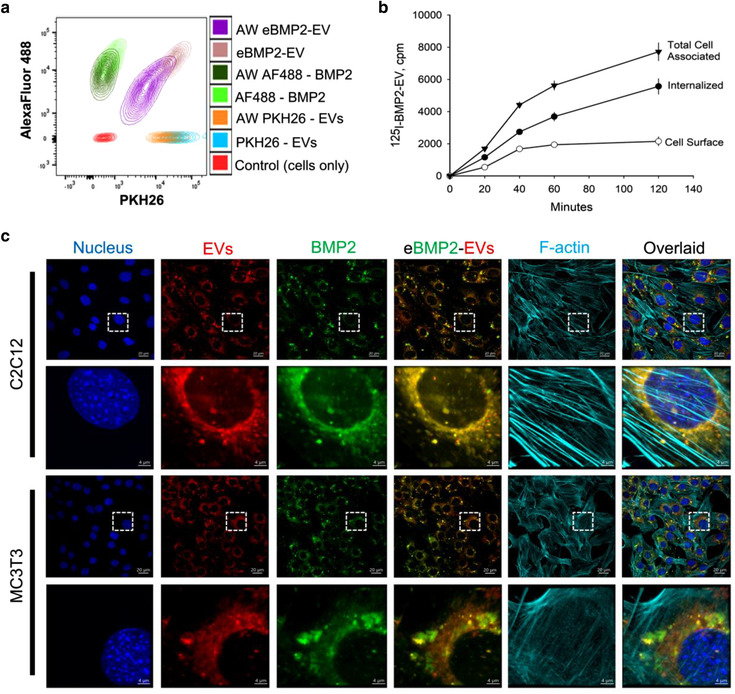
In vitro assessment of cellular uptake and signalling by liquid‐phase eBMP2‐EVs. (a) Flow cytometric analysis of cellular uptake of BMP2, EVs and eBMP2‐EVs by MC3T3 cells at 4 h with and without acid washing (AW). BMP2 was labelled with Alexa Fluor 488 (AF; green) and EVs were labelled with PKH26 (red). (b) Cellular uptake of ^125^I‐eBMP2‐EVs by C2C12 cells. (c) Representative confocal images showing cellular uptake of fluorescently labelled eBMP2‐EVs by C2C12 and MC3T3 cells after 6 h. Nucleus (blue), EV (red), BMP2 (green) and F‐actin (indigo)

Both fluorescence and radioactive labelling were used to determine cell internalization of eBMP2‐EVs. By example, Alexa Fluor 488‐BMP2 was loaded into EVs to determine cell uptake by MC3T3 cells using flow cytometry and fluorescence microscopy, and ^125^I‐BMP2 was loaded into EVs to evaluate eBMP2‐EV uptake in C2C12 cells. Acid wash post BMP2 loading ensured that BMP2 was restricted to the EV lumen. Flow cytometry demonstrated Alexa Fluor 488‐eBMP2‐EVs internalization into MC3T3 cells (Figure 2a). Alexa Fluor 488‐BMP2 uptake was also evaluated in C2C12 cells. At 4 h, cells were analysed for internalization of BMP2, EVs and eBMP2‐EVs. In the contour plot shown in Figure 2a, the X‐axis indicates the PKH26 fluorescence (EV‐label) while the Y‐axis indicates the AlexaFluor 488 fluorescence (BMP2‐label). For each, BMP2, EVs and eBMP2‐EVs, approximately 30% was surface bound to MC3T3 cells at 4 h, which was washed‐off during acid rinsing. When ^125^I‐eBMP2‐EVs were incubated with C2C12 cells, cell surface‐bound ^125^I‐eBMP2‐EVs became saturated within 45 min, whereas internalized ^125^I‐eBMP2‐EVs continued to accumulate inside the cell over the 2 h incubation period (Figure 2b). All subsequent radioactive cell binding experiments were based on a 1 h incubation. Confocal microscopy (Figure 2c) revealed that the internalized eBMP2‐EVs accumulated in the cytoplasm and were concentrated perinuclearly for both C2C12 and MC3T3 cells. Overlay images at higher magnification indicated intact eBMP2‐EVs, as well as ‘free’ BMP2 alone and EVs without BMP2, suggesting in part the release of ‘free’ BMP2 into the cytoplasmic compartment. Additional 3D confocal Z‐stack movies are provided in the supplemental section, movie S1 and movie S2.

### 3.3 eBMP2‐EVs induce osteoblastic differentiation

**FIGURE 3:**
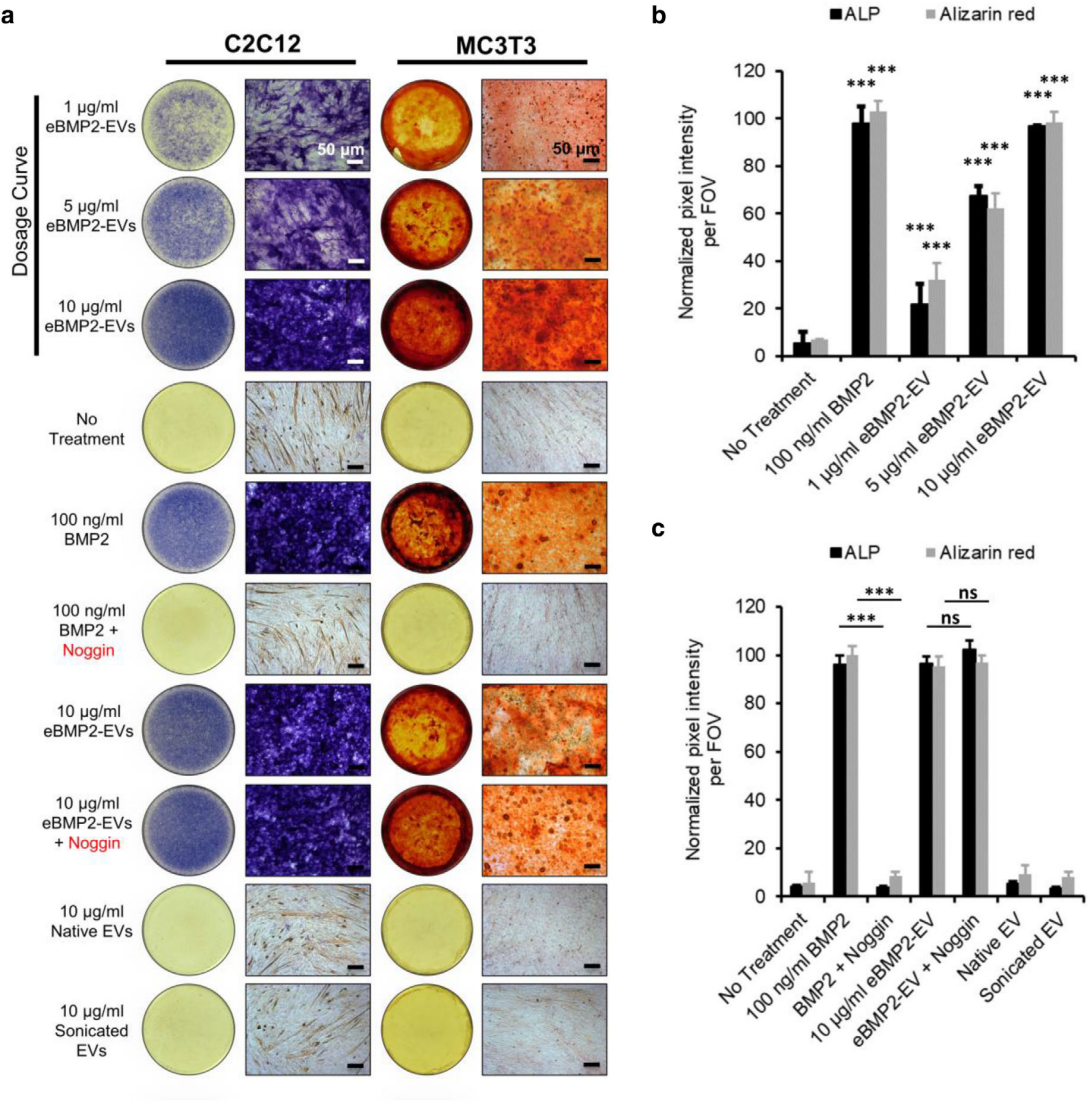
In vitro assessment of liquid‐phase eBMP2‐EVs bioactivity (a) Bright‐field images of ALP assay (C2C12 cells) and mineralization assay (MC3T3 cells). Shown is a representative experiment of 3 independent experiments (b) Dosage curve of eBMP2‐EV's bioactivity in ALP assay and mineralization assay. Data represents the mean and SEM for 3 independent experiments; *** = ρ≤0.001; ns = no significant difference vs the no treatment control group. (c) Quantification of ALP staining and alizarin red staining for indicated treatments. Data represents the mean and SEM for 3 independent experiments; *** = ρ≤0.001; ns = no significant difference vs the noggin control group

eBMP2‐EVs applied in the liquid‐phase induced cytological markers for osteoblast differentiation. An early osteogenic marker, ALP, was accessed in C2C12 cells. Mineralization, a late osteogenic marker, was accessed in MC3T3 cells using alizarin red staining for calcium. Both ALP and mineralization were induced by eBMP2‐EVs (Figure 3a). Neither native non‐sonicated or sonicated EVs (without BMP2) induced osteogenic differentiation. Following the quantitation of cytological staining, a dose‐dependency for both ALP and mineralization was demonstrated (Figures 3b and 3c). The highest dose of eBMP2‐EV (180 ng BMP2 in 10 μg EV protein) resulted in similar stimulation of both osteogenic markers compared to the 100 ng BMP2 positive control group.

### 3.4 EV intraluminal BMP2 is no subject to noggin inhibition of osteoblastic differentiation

Soluble ‘free’ BMP2 within the extracellular microenvironment is subject to binding to various BMP2 antagonist proteins that block BMP2 interaction with its cell surface receptors, thus disrupting BMP2 signalling. Because eBMP2‐EVs contain BMP2 within the EV lumen, we sought to determine if a BMP2 sequestering protein, noggin, could disrupt eBMP2‐EV stimulation of osteoblast differentiation. The stimulation of both ALP and mineralization by eBMP2‐EVs was found not to be inhibited by 1 μg/ml noggin, whereas ‘free’ BMP2‐mediated differentiation was inhibited (Figures 3a and 3c). These data demonstrate that BMP2 contained within the luminal compartment of EVs is protected from the inhibitory interaction with extracellular binding inhibitors, such as noggin.

### 3.5 Cell trafficking of BMP2 in C2C12 cells

Cell trafficking experiments including recycling of cell membrane internalized growth factors has been extensively studied in regard to ^125^I‐epidermal growth factor (EGF), but to our knowledge EV or BMP2 cell recycling has been either minimal or not considered. We measured the C2C12 association and internalization of ^125^I‐BMP2 and its subsequent release into the extracellular compartment (Table 1). After 1 h at 37°C, cell bound BMP2 was represented as ∼20% of ^125^I BMP2 associated with the cell surface while the remaining represented cell internalized ^125^I BMP2. The cell surface associated radioactivity represented BMP2 bound to both BMP2 receptors and heparin sulfate proteoglycans (HSPGs), with HSPG binding sites being the predominant type in C2C12 cells38.

**TABLE 1:** Cell trafficking of ^125^I‐BMP2 in C2C12 cells

Total Cell Associated BMP2	
Cell Surface %	19.68 + 0.72
Internalized %	80.32 + 0.72
Recycled BMP2	
Total Recycled, % Internalized	40.36 + 0.50
Cell Surface %	13.78 + 0.57
Released to Media %	86.22 + 0.57
Recycled Media BMP2	
EV fraction %	82.23 + 0.58
“Free” BMP2 fraction %	13.54 + 0.56
Recycled Media BMP2 EV, cargo location	
EV surface %	92.01 + 0.77
EV lumen %	7.99 + 0.77

Data represent the mean + SEM of 6 replicate cultures of a representative experiment for total cell associated BMP2 and recycled BMP2. Whereas data represents the mean + SEM of 3 experiments conducted using ‘pooled’ recycled media BMP2.

After acid stripping of cell surface bound ^125^I‐BMP2 and following the return of stripped cells to fresh binding media at 37 °C, recycling of internalized ^125^I‐BMP2 was determined. A total of ∼40% of internalized ^125^I‐BMP2 was externalized with ∼14% associated with the cell surface and the remaining released into the media. When the recycled media BMP2 was eluted using SEC, ∼82% of the ^125^I‐BMP2 eluted as ^125^I‐BMP2‐EVs which we designated ^125^I‐nBMP2EVs. The remaining recycled media BMP2 represented ‘free’ BMP2.

To determine the location of BMP2 as EV cargo, ^125^I‐nBMP2‐EVs were treated with trypsin, 37°C, 30 min, followed by SEC elution. Compared to non‐trypsin control groups, trypsin treatment resulted in ∼92% loss of ^125^I‐BMP2 from the EV fraction, suggesting that the bulk of BMP2 associated as EV cargo in nBMP2‐EVs was bound to the surface. The remaining 8% was designated as intralumenal BMP2. Additional experiments were performed to access the ability of heparin to disassociate ^125^I‐BMP2 from ^125^I‐nBMP2‐EVs. These experiments indicated that heparin treatment of ^125^I‐nBMP2‐EVs for 15 min, 23°C, resulted in a 90% + 0.12% (mean + SEM, 3 independent experiments) loss of ^125^I‐BMP2 from the EV fraction, following SEC elution. This further supports that BMP2 in nBMP2‐EVs is associated with the EV surface, and likely bound to glycan groups, such as HSPG on the EV surface or other EV surface proteins via similar electrostatic association.

### 3.6 Recycling of cell internalized BMP2 and the generation of nBMP2‐EVs

**FIGURE 4:**
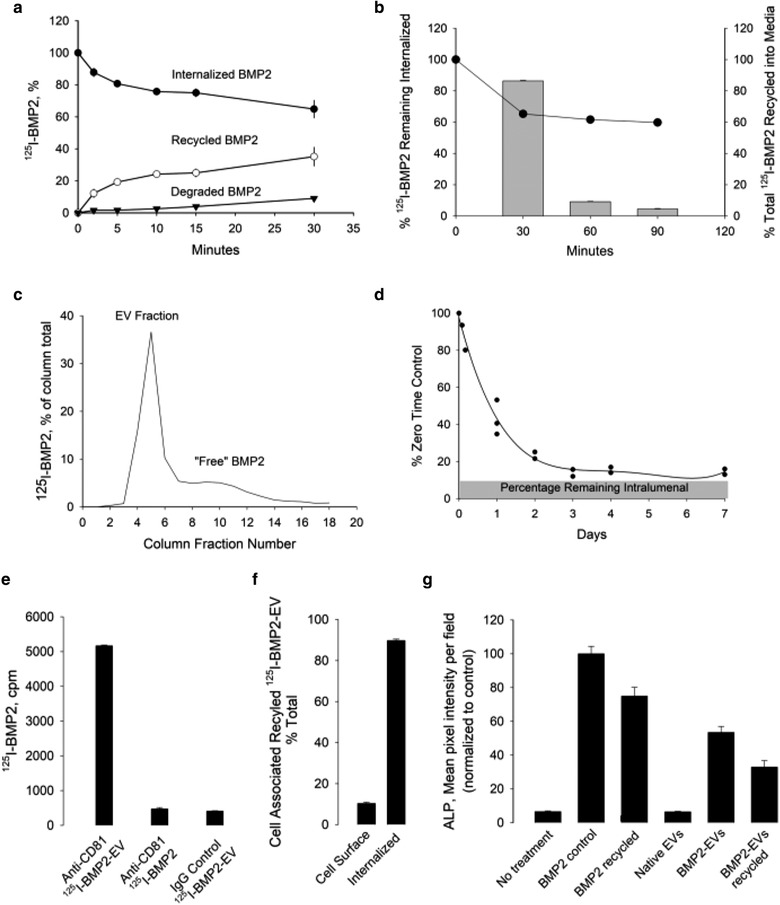
Recycling of BMP2 as nBMP2‐EVs from C2C12 cells. (a) Temporal release of internalized ^125^I‐BMP2 at 37 °C. (b) Bulk of ^125^I‐BMP2 release occurs by 30 min, 37 °C. (c) Assessment of recycled ^125^I‐BMP2 after elution over 2B Sepharose SEC. Fractions 4‐6 represented ^125^I‐nBMP2‐EV while fractions 9‐11 represented “free” ^125^I‐BMP2. (d) Retention of ^125^I‐BMP2 on the EV surface under simulated in vitro body conditions. ^125^I‐nBMP2‐EVs were incubated at 37 °C and at indicated timepoints samples were eluted over 2B Sepharose SEC to determined ^125^I‐BMP2 remaining bound to EV fraction. Cell surface and released distribution of recycled ^125^I‐BMP2‐EVs. Middle panel Recycling of ^125^I‐BMP2. (e) Immunoprecipitation of recycled ^125^I‐BMP2‐EV. Aliquots (∼7000 cpm) of recycled ^125^I‐BMP2‐EV or ^125^I‐BMP2 control were immunoprecipitated with either 25 μg of anti‐CD81 or with IgG. (f) Cell association of ^125^I‐nBMP2‐EV after 1 h, 37 °C. (g) Osteogenic activity of recycled BMP2. Unlabelled BMP2 was incubated with C2C12 cells and the 30 min recycled media was collected, pooled, concentrated and eluted over 2B Sepharose SEC. The protein concentration for EV and BMP2 fractions was determined and aliquots of each were assessed for alkaline phosphatase activity compared to eBMP2‐EV and control BMP2. Shown is a representative experiment with bars representing mean + SEM for 3 or 6 replicates

Internalized ^125^I‐BMP2 exhibited recycled release into media in as little as 2 min with minimal degradation throughout the sampling period (Figure 4a). Recycling occurred in essentially the first 30 min with minimal cell release occurring afterwards (Figure 4b) with ∼60% remaining internalized. The recycled media ^125^I‐BMP2 was pooled and samples eluted over SEC to establish distribution between EV and ‘free’ BMP2 fractions (Figure 4c). We assessed the stability of BMP2 on the surface of ^125^I‐nBMP2‐EVs was similar to ^125^I‐eBMP2‐EVs in Figure 1e. In direct contrast to intralumenal BMP2 in eBMP2‐EVs, BMP2 associated with the surface of nBMP2‐EVs became dissociated from the surface under simulated in vivo conditions (Figure 4d). Essentially 99% of the EV surface BMP2 associated with nBMP2‐EVs was released after 3 days, suggesting on‐going dissociative release of ‘free’ BMP2 from nBMP2‐EVs. Such a delayed release of BMP2 from EVs could provide an extended delivery of ‘free’ BMP2 to bind to its cell surface receptors. This is beyond the likely direct binding of BMP2, while on the surface of nBMP2‐EVs, to BMP2 cell surface receptors.

Soluble ^125^I‐BMP2 and unlabelled BMP2 were derived from recycling experiments and evaluated as a ‘naturally’ occurring, cell‐derived form of BMP2‐EV. Experiments were designed to answer the question is internalized ‘free’ BMP2 recycled into biologically functional BMP2‐EVs? Immunoprecipitation of ^125^I‐nBMP2‐EVs using anti‐CD81 antibodies confirmed that the recycled nBMP2‐EV fraction likely corresponds to exosome subfraction of EVs (Figure 4e). ^125^I‐nBMP2‐ EV, representing recycled BMP2 into EVs by C2C12 cell trafficking, represents ∼66% of the soluble recycled fraction. This cell‐derived form of BMP2‐EV, was capable of binding to and being internalized into C2C12 cells (Figure 4f). Biological activity of both recycled nBMP2‐EV and ‘BMP2’ fractions was demonstrated using ALP assay (Figure 4g). Similar overall protein concentrations of BMP2‐EVs at 25 μg/ml, both sonication loaded and recycled loaded, and ‘free’ BMP2 at 100 ng/ml, both stock BMP2 and recycled BMP2, all resulted in significant ALP expression. The slightly lower response for both recycled nBMP2‐EV and recycled ‘free’ BMP2 compared to their respective control groups most likely reflects other protein constituents recycled along with nBMP2‐EVs and ‘free’ BMP2 contributing the total protein concentration, but also that loading differences between EV preparations do not lend themselves to direct concentration comparisons.

### 3.7 Cell trafficking of eBMP2‐EVs in C2C12 cells

Expanding on the cell internalization experiments presented in Figure 2, ^125^I‐eBMP2‐EVs were incubated with C2C12 cells to indicate both internalization and recycling. After 1 h, 37°C, ∼74% of ^125^I‐eBMP2‐EVs were internalized within cells, the remainder on the cell surface (Table 2). After the return of acid‐rinsed cells to culture, after 30 min ∼24% of the internalized ^125^I‐eBMP2‐EVs were recycled with ∼83% of the recycled ^125^I‐eBMP2‐EVs being released into the media and the reminder associated with the cell surface. The remaining ∼76 % of ^125^I‐BMP2 was retained intracellularly. The cell surface associated radioactivity represents EV membrane ‘receptors’, such as HSPGs, and likely represent receptors existing on the cell surface at the time of the initiation of the recycling incubation and/or ‘receptors’ that have been recycled along with ^125^IeBMP2‐EVs. The recycled media ^125^I‐eBMP2‐EVs were of insufficient radioactivity, even with pooling to evaluate EV and ‘free’ BMP2 distributions. However, using directly radiolabeled ^125^|‐EVs, we determined that cell trafficking of eBMP2EVs was similar to EVs alone, with the majority of the recycled media EVs being associated the EV fraction (data not shown), suggesting recycling of intact EVs. However, whether these recycled EVs represent exocytosis of unmodified EVs or EVs modified during endosomal processing, remains unknown.

**TABLE 2:** Cell trafficking of ^125^I‐eBMP2‐EVs in C2C12 cells

Total Cell Associated eBMP2‐EVs	
Cell Surface %	25.09 + 1.02
Internalized %	74.09 + 1.02
Recycled BMP2	
Total Recycled, % Internalized	23.81 + 1.00
Cell Surface %	17.35 + 0.66
Released to Media %	82.65 + 0.66

Data represent the mean + SEM of 4 experiments.

### 3.8 Comparing osteogenic gene and protein expression induced by ‘free’ BMP2 and eBMP2‐EVs

The data above demonstrate not only the internalization of eBMP2‐EVs but also that the osteogenic potential of eBMP2‐EVs is not inhibited by noggin. The absence of EV surface BMP2 and the lack of inhibition of EV intralumenal BMP2 suggest that BMP2 is internalized directly as EV intralumenal cargo, bypassing any interaction with its cell surface BMP2 receptors. This suggests that BMP2 signalling is directly initiated intracellularly. Therefore, we looked at various cytoplasmic molecular players associated with the BMP2 signalling pathway in both C2C12 and MC3T3 cells, to determine if there were differences between BMP2 signalling cascades initiated by cell surface receptors and presumably cytoplasmic receptors.

**FIGURE 5:**
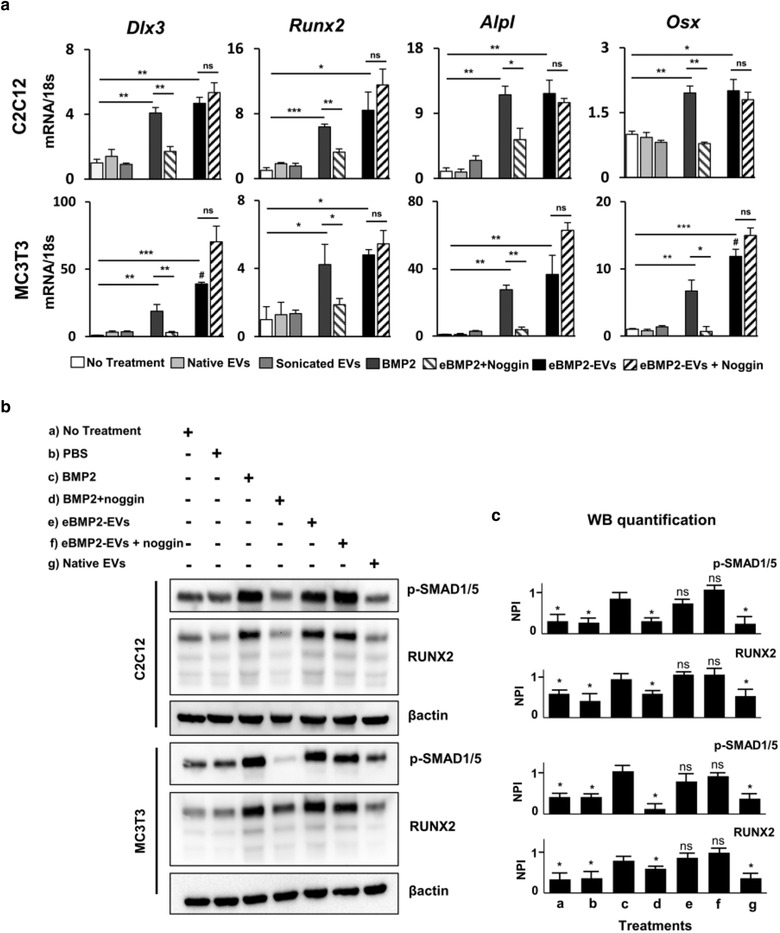
In vitro comparison of liquid‐phase BMP2 and eBMP2‐EV signalling. (a) qPCR mRNA expression profiles for Dlx3, Runx2, Alpl, and Osx in C2C12 and MC3T3 cells subjected to indicated treatments for 72 h. Shown is a representative experiment for 2‐3 independent biological experiments with error bars indicating SEM for 3 biological replicates. (* = ρ≤0.05, ** = ρ≤0.01, *** = ρ≤0.001 between compared groups, # = ρ≤0.01 vs BMP2 group) (b) C2C12 and MC3T3 were subjected to indicated treatments for 48 h and western blot analysis for phospho‐SMAD1/5, RUNX2 and β‐actin protein expression was evaluated. (c) Relative quantitation of western blot image (Figure 4b) band intensities relative to β‐actin. NPI: Normalized pixel intensity, treatments (a‐g) corresponds to respective treatments in (c). Bars indicate mean ± SEM (3 independent experiments), * = ρ≤0.05 vs BMP2 group

Several homeodomain (HD) proteins are critical for skeletal patterning and respond directly to BMP2 as an early step in bone formation. RUNX2, the earliest transcription factor proven essential for commitment to osteoblastogenesis, is also expressed in response to BMP2. The first set of experiments compared cell surface initiated BMP2 (100 ng/ml) and cytoplasmic initiated eBMP2‐EVs (180 ng BMP/10 μg/ml EV protein), both in the absence or presence of 1 μg/ml noggin, for their ability to induce the expression level of transcripts of osteogenic genes *Dlx3*, *Runx2*, *Alpl* and *Osx* after 48 h post‐stimulation, as evaluated using qRT‐PCR. These genes were selected given prior reports showing their involvement in BMP2‐induced osteogenic differentiation. (Hassan et al., 2006) Both BMP2 and eBMP2‐EVs induced osteogenic gene expression for all four genes for both C2C12 and MC3T3 cells (Figure 5a). In both the cell lines, native EVs and sonicated EVs had no effect on any osteogenic genes suggesting that J774A.1 EVs do not carry native osteogenic cargo. Both BMP2 and eBMP2‐EVs resulted in multi‐fold increase in levels of all the osteogenic genes in both cell lines compared to no treatment control (Figure 5a). *Alpl* had the most drastic (12‐fold) increase in presence of BMP2 and eBMP2‐EVs. In C2C12s there was no difference in the expression levels of all the four genes under BMP2 or eBMP2‐EV stimulation. However, in MC3T3 cells, there was 20% higher expression of *Dlx3* and *Osx* under eBMP2‐EV treatment compared to BMP2 treatment. Given this is only a 0.2‐fold expression difference, this may or may not hold much significance. The multi‐fold increase in expression levels of all the four genes was inhibited when noggin was added along with BMP2. However, noggin did not inhibit the induction of osteogenic genes by eBMP2‐EVs. These data suggest that in addition to ALP and mineralization, osteogenic gene upregulation is similarly stimulated by both BMP2 and eBMP2‐EVs. Furthermore, similar to ALP and mineralization experiments, while noggin blocked gene expression for all genes, it failed to inhibit the corresponding osteogenic gene expression induced by eBMP2‐EVs.

Previous reports show that the association of RUNX2 and SMAD1/5 is essential for BMP2‐induced osteogenic induction of C2C12 cells and that the physical interaction between RUNX2 and SMADs is dependent on ERK‐mediated phosphorylation of RUNX2. (Afzal et al., 2005) Therefore, western blotting experiments were performed to evaluate the protein expression of pSMAD 1/5 and RUNX2 in both MC3T3 and C2C12 cells stimulated by BMP2 and eBMP2‐EVs. After 72 h exposure to BMP2 treatments, proteins were extracted for western blot analysis. Both BMP2 and eBMP2‐EVs increased the protein expression of pSMAD 1/5 and RUNX2 in both cell types (Figures 5b and 5c). Similar to gene expression experiments, while noggin blocked the upregulation of pSMAD 1/5 and RUNX2 by BMP2, noggin did not inhibit the effect of eBMP2‐EVs (Figures 5b and 5c). Full blot images are shown in Figures S2 and S3.

**FIGURE 6:**
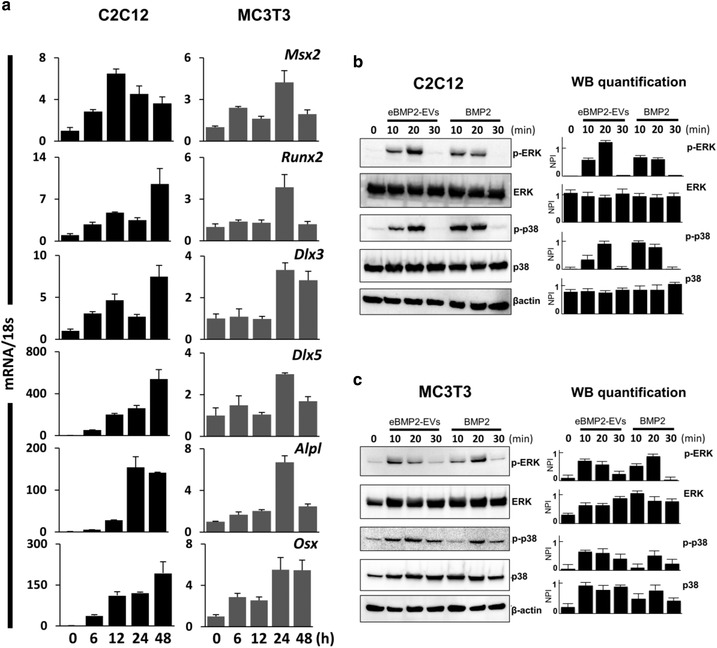
In vitro assessment of liquid‐phase eBMP2‐EV signalling kinetics. (a) qPCR mRNA expression profiles for Msx2, RunX2, Dlx3, Dlx5, Alpl, and Osx in C2C12 and MC3T3 cells treated with eBMP2‐EVs for indicated time‐points. (b‐c) C2C12 and MC3T3 cells were treated with BMP2 or eBMP2‐EVs for indicated time points and levels of total and phosphorylated ERK/p‐ERK, p38/pp38 and β‐actin were determined using western blot. Graphs next to the western images represent quantitated band intensities relative to β‐actin. Bars indicate mean ± SEM (3 independent experiments), NPI: Normalized pixel intensity

Lastly, a set of gene and western blot kinetic experiments was conducted to compare the temporal effects of BMP2 and eBMP2‐EVs on the gene expression of *Msx2, Runx2, Dlx3, Dlx5, Alpl* and *Osx. e*BMP2‐EVs induced time‐dependent gene expression of all transcription genes in both cell types (Figure 6a). These results agree with the literature where ‘free’ BMP2 is reported to stimulate the expression of these transcription factors in both C2C12 and MC3T3 cells (Celil et al., 2005; Cheng et al., 2003; Hassan et al., 2006; Lee et al., 2003). In C2C12 cells *Msx2* peaked in 6 h following which it went down, whereas in MC3T3 cells it showed a bimodal expression pattern with first peak in 6 h followed by the second one in 24 h. *Runx2* and *Dlx3* had bimodal expression patterns in C2C12s with first peak in 12 h and a second peak appearing in 48 h. In MC3T3 cells, *Runx2* and *Dlx3* were stimulated after 24 h. *Dlx5* was stimulated in 48 h in C2C12 cells, while it showed a bimodal expression level in MC3T3 cells with a small peak in 6 h and a second peak around 24 h beyond which it went down. In both the cell lines, *Alpl* expression peaked in 24 h after treatment but remained elevated only in C2C12s while it fell to normal levels in MC3T3 cells in 48 h. On the other hand, *Osx* expression appeared to peak in 48 h in C2C12 cells while it peaked in 24 h in MC3T3 cells.

BMPs can signal through both canonical and non‐canonical pathways. In the canonical signalling pathway, they initiate the signal transduction cascade by binding to cell surface receptors resulting in phosphorylation of SMAD. The phosphorylated SMAD proteins are then translocated to the nucleus, where they bind specific motifs in promoter regions, recruit RUNX2, and regulate the transcription of target genes. Several non‐canonical, SMAD‐independent signalling pathways for BMPs have been identified of which MAPK cascades are one of the most studied. In the non‐canonical MAPK pathways, BMP2 activates the p38, ERK and JNK1/2 signalling pathways to promote the expression and activation of RUNX2. To compare the temporal effects of BMP2 and eBMP2‐EVs on non‐canonical BMP signalling pathways, phosphorylation of ERK and p38 proteins was assessed. The phosphorylation of ERK and p38 was similarly stimulated for both BMP2 and eBMP2‐EVs, with phosphorylation occurring within 10 min, then reducing or ceasing by 30 min for both cell types (Figures 6b and 6c). Full blot images are shown in Figures S4 and S5. Overall, the gene and protein expression levels of transcription factors involved in canonical and non‐canonical BMP signalling pathways were upregulated via both BMP2 and eBMP2‐EVs, which supports a broad overlap in osteogenic signalling whether initiated at the cell surface or from the cytoplasm.

### 3.9 Bioprinted eBMP2‐EVs microenvironments induce osteoblastogenesis in registration to printed patterns

**FIGURE 7:**
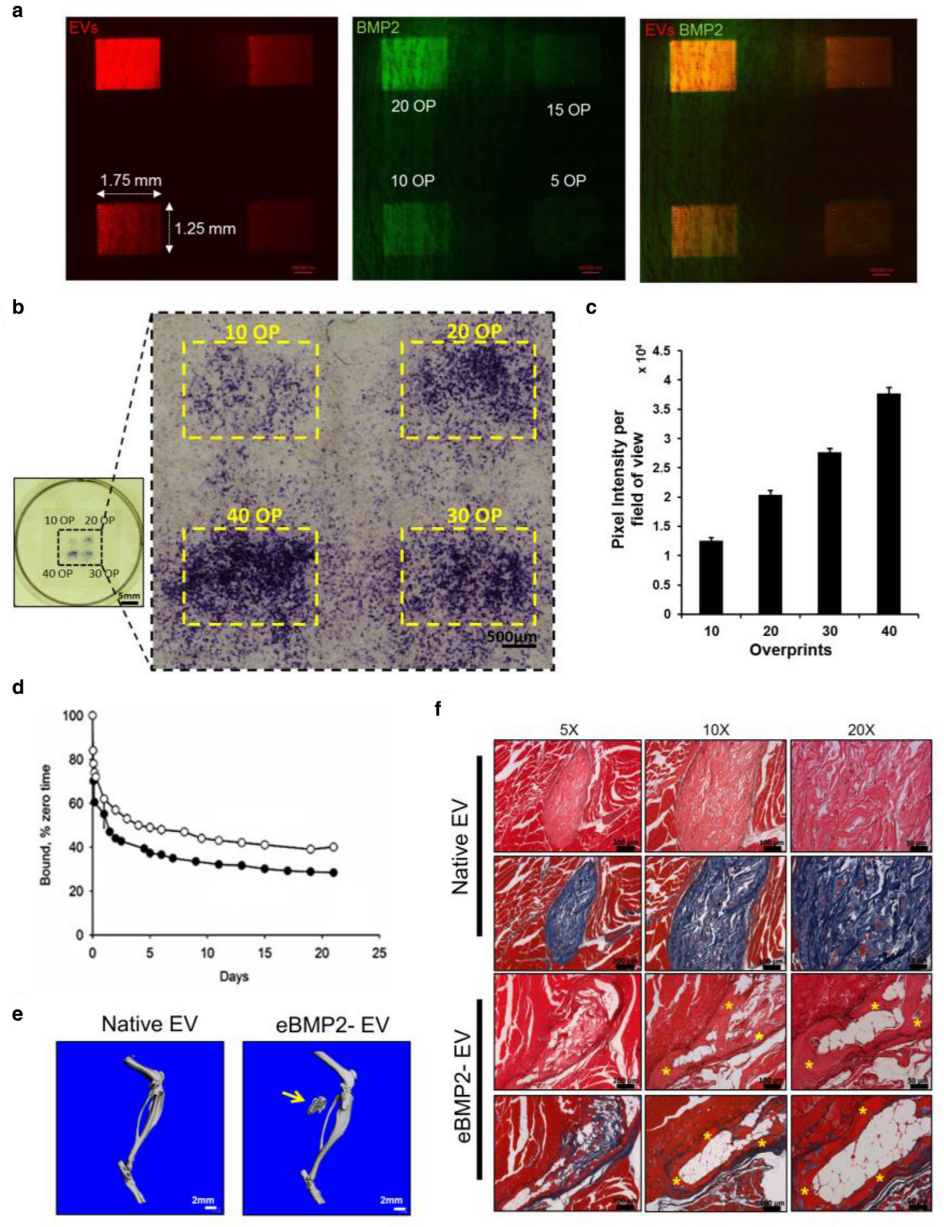
In vitro and In vivo assessment of solid‐phase BMP2‐EVs microenvironments. (a) Bioprinted patterns of Alexa Fluor 488‐labeled BMP2 (green) loaded in PKH26‐labeled EVs (red). (b) ALP staining of C2C12s post 72 h seeding on bioprinted patterns with indicated OPs. (c) Quantification of ALP staining of bright‐field images shown in Figure 7b. Shown is a representative experiment for 5 independent biological experiments with error bars indicating SEM. (d) Release kinetics of ^125^I‐BMP2EVs from ADM in simulated body fluid for 3 weeks. (e) Representative μCT 3D reconstructions of mouse leg scans containing either native EV or BMP2‐EVs bioprinted implants. Arrow points to HO. (f) Representative histological images showing H&E and Masson's trichrome staining of native EVs and BMP2‐EVs bioprinted implants (*indicates bone tissue)

Patterns of eBMP2‐EVs (Alexa Fluor 647‐labeled BMP2 and PKH26‐labeled EVs) were printed onto collagen type I coated coverslips and imaged for fluorescence as shown in Figure 7a. The presence of fluorescence from BMP2 and EVs after overnight rinsing in PBS demonstrated successful bioprinting and retention of eBMP2‐EVs on the collagen type I coated coverslips. For in vitro biological response experiments, C2C12 cells were cultured on bioprinted eBMP2‐EV microenvironments for 3 days before staining for ALP response. C2C12 cells associated with printed patterns of eBMP2‐EVs differentiated toward the osteogenic lineage as evidenced by increased ALP activity (Figure 7b). ALP responsiveness was in registration to the printed BMP2‐ EVs patterns and demonstrated an increase in ALP with increasing deposited concentrations of eBMP2‐EVs (Figure 7c). Limited ALP response did occur off pattern immediately adjacent to pattern edges, and the intensity the off‐pattern ALP response appeared directly proportional to deposited eBMP2‐EV concentrations, although the vast majority of cells associated outside the spatially defined eBMP2‐EVs patterns did not exhibit appreciable ALP activity and thus remained undifferentiated.

### 3.10 Bioprinted BMP2‐EVs constructs locally induce heterotopic ossification (HO)

2 × 2 mm ADM scaffolds were bioprinted with ^125^I‐BMP2, ^125^I‐EVs or ^125^I‐eBMP2‐EVs and their binding retention was evaluated over 3 weeks in SBF as shown in Figure 7d. After 3 weeks ∼40% of ^125^I‐BMP2 was retained in ADM whereas ∼30% of ^125^I‐EV and ^125^I‐eBMP2‐EVs were retained in ADM. Both, native EVs and eBMP2‐EVs followed a similar release profile that was different from ‘free BMP2’. Printed eBMP2‐EV constructs (5 ng deposited BMP2 concentration per 4.5 mm circular ADM construct) directed spatially controlled HO formation when implanted in a murine muscle pocket for 4 weeks. Radiographic μCT evaluation (Figures 7e, 7f and S6) demonstrated spatially controlled HO formation at the site of eBMP2‐EV construct implantation while the native EV printed ADM control construct did not. Following radiographic analysis, we performed decalcified bone histology on implant sites containing constructs and confirmed that sites of radiographic HO formation also represented histological bone (Figure 7e) as identified by H&E and Masson's trichrome staining for cells and bone morphology based on organized deposited organic matrix constituents. Decalcified histology was selected as a standard approach providing superior cell level microscopic resolution compared to undecalcified histology (Kang et al., 2003) and represents our standard approach for bone histological analysis (Brooker et al., 2019; Cooper et al., 2010a, 2010b; Tuzmen et al., 2018).

## 4 DISCUSSION

In this study we developed and characterized eBMP2‐EVs, and as a result propose that they signal cells by a novel mechanism. We further describe how BMP2 cell signalling involves complex cell trafficking of BMP2, including the extracellular recycling of not only bioactive ‘free’ BMP2, not associated with EVs, but also BMP2 bound to the surface of secreted EVs as nBMP2‐EVs. nBMP2‐EVs present BMP2 on the EV surface exhibiting similar cell signalling and noggin inhibition as observed for ‘free’ BMP2. Whereas, eBMP2‐EVs do not contain EV surface BMP2, therefore the intralumenal BMP2 is unable to directly interact with cell surface BMP2 receptors. However, despite its intraluminal BMP2 location, eBMP2‐EVs regulated in vitro osteoblastogenesis gene/protein expression, as well as in vitro mineralization, similarly to ‘free’ BMP2. Furthermore, eBMP2‐EV regulation of osteoblastogenesis was protected from its inhibitor noggin for up to 1 month in cell culture. Lastly, eBMP2‐EVs can also occur in the solid‐phase (immobilized in ECM) and regulate osteoblastogenesis in register to its deposited pattern.

Macrophages play an important role in osteogenic differentiation and EVs from macrophages have been utilized to deliver exogenous biomolecules (Haney et al., 2015; Sinder et al., 2015). Therefore, we decided to use murine J774A.1 cells as our source of EVs. To study osteoblastic differentiation in vitro, we utilized C2C12 and MC3T3 cells. MC3T3 represents a pre‐osteoblastic origin cell line, whereas C2C12, although of myogenic origin, essentially represents a mesenchymal stem cell line. Therefore, the use of both cell lines complements each other. MC3T3 is already committed to an osteoblastic lineage, whereas C2C12 must be induced by BMP2 into an osteoblastic lineage. We selected C2C12 cells for ALP experiments because MC3T3 express a basal level of ALP in comparison to C2C12 cells, whereas MC3T3 typically give us more definitive mineralization results (Kassick et al., 2018; Phillippi et al., 2008; Tuzmen & Campbell, 2018). In developing eBMP2‐EVs, sonication of EVs in the presence of BMP2 provided the greatest loading capacity which has been prior reported as an efficient EV loading strategy for proteins (Haney et al., 2015). Although the data suggested a possible ∼30 % reduction in EVs post sonication (Figure 1b), we stress that tunable resistive pulse sensing is not optimized to accurately evaluate EV concentration. More importantly, the overall size distribution profile of sonicated EVs or eBMP2‐EVs (EVs sonicated in presence of BMP2) remained unchanged as compared to native EVs (non‐sonicated EVs). Sonication‐based loading of BMP2 into eBMP2‐EVs was estimated at 18 ng BMP2/μg EV protein. This concentration of BMP EV loading far exceeds that of naturally occurring BMP2 in EVs isolated from rat growth plates at 121 pg BMP2/μg EV protein (Garimella et al., 2004; Nahar et al., 2008). We re‐emphasize here that the BMP2 is intralumenal in eBMP2‐EVs, whereas essentially all of the BMP2 is EV surface associated in naturally occurring EVs either reported here as nBMP2‐EVs or in EVs secreted during embryotic development (Draebing et al., 2018). In addition, we loaded BMP2 into preformed EVs that are neutral in regard to osteogenesis (Figure 3c, Figure 4, Figure 7), therefore, there is no confounding of the BMP2 osteogenic effect with other osteogenic signalling aspects such as reported in Huang *et. al*. (Huang et al., 2020). Furthermore, while Huang *et. al*. demonstrated that EVs derived from cells engineered to overexpress BMP2 were osteogenic, they found the EVs did not contain BMP2 (Huang et al., 2020).

Currently, BMP2 signalling is considered to be initiated upon BMP2 complexing to cell surface BMP receptors type I and II initiating intracellular signal signalling cascades via SMAD‐dependent (canonical) and MAPK SMAD‐independent (non‐canonical) pathways that regulate osteogenic gene transcription (Majidinia et al., 2018). Internalization of the signalling BMP2‐BMPR complex occurs via clathrin and caveolin endocytosis (Bonor et al., 2012), although BMP2 signalling can persist without internalization (Hauff et al., 2015; Pohl et al., 2012). Upon endocytosis, the signalling BMP2‐BMPR complex is incorporated into endosomes where it continues to signal intracellularly. Endosomal processing continues with either recycling of BMP2 (Figure 4c) and BMPR (Gleason et al., 2014), or eventual degradation within the lysosome. Presumably, cell signalling of nBMP2‐EVs is also initiated via direct interaction of EV surface BMP2 with cell surface BMP2 receptors (Draebing et al., 2018) and as suggested by our data here. Alternatively, HSPG also acts as a cell surface BMP2 binding ‘receptor’ moiety. In C2C12 cells, HSPG BMP2 ‘receptors’ far exceed traditional BMP2 receptor cell surface populations and compete for BMP2‐BMP receptor complex formation and subsequent cell signalling (Jiao et al., 2007; Takada et al., 2003), HSPG is involved in cell internalization of BMP2 and reported to act as a sink to remove extracellular ‘free’ BMP2 (Alborzinia et al., 2013; Jiao et al., 2007; Kim et al., 2021; Takada et al., 2003).

To our knowledge recycling of cell internalized BMP2 has not been considered, although BMP2 direct cell‐to‐cell transcytosis has been suggested (Alborzinia et al., 2016). Our data suggest the release of bioactive ‘free’ BMP2 and nBMP2‐EVs (Figure 4c) to the extracellular environment. Upon extracellular release, recycled ‘free’ BMP2 is subject to immediate reassociation to cell surface BMP2 receptors or to HSPG, as well as its release into the interstitial liquid environment or immobilization within the ECM. BMP2 recycled as nBMP2‐EVs can be released into the interstitial liquid environment, become immobilized within the ECM, bind to BMP2 receptors either as ‘free’ BMP2 dissociated from nBMP2‐EVs, as nBMP2‐EVs, or bind to cell surface HSPG. Furthermore, recycling of the BMP2 receptors (Hartung et al., 2006), HSPG (Christianson & Belting, 2014) and noggin (Phan‐Everson et al., 2020) has been proposed.

BMP2/4 are inhibited by noggin by disrupting BMP access to its cell surface BMPRs (Mcmahon et al., 1998), Additionally, noggin also promotes the cellular internalization of non‐signalling BMP2 (Alborzinia et al., 2013; Liang et al., 2020). This involves the formation of a binding complex of BMP‐noggin‐HSPG ‘receptors’ on the cell surface (Paine‐Saunders et al., 2002), which internalize via HSPG (Christianson & Belting, 2014). After internalization into perinuclear endosomes, acid‐stable binding of noggin to BMP2 results in continued inhibition of BMP bioactivity (Alborzinia et al., 2016). Whereas, upon internalization of BMP2 directly via HSPG, without noggin and with extracellular BMP2 removed from culture post‐internalization, there is an eventual loss of internalized BMP2 which in part has been attributed to the direct transfer to immediate neighbouring cells via vesicular transcytosis processes (Alborzinia et al., 2016).

Considering our data, it is possible that BMP2 internalized via HSPG is recycled as both ‘free’ BMP2 and nBMP2‐EVs which may in part be responsible for paracrine signalling of neighbouring cells beyond transcytosis. In the continued presence of BMP2 in the incubation media, BMP2 is internalized and remains intact (Alborzinia et al., 2013; Kim et al., 2021). However, as these other reports did not consider BMP2 recycling it is unclear whether the internalized BMP2 represents either static loading or continuous loading, recycling and reloading. Since we only considered early loading in this study, our focus will shift to longer incubation and recycling conditions, as well as the impact of noggin, in future studies. Recycling of noggin and its involvement in intracellular BMP2 signalling also remains to be determined. Beyond BMP2‐noggin‐HSPG complexes with noggin serving as the binding intermediary between BMP2 and HSPG (Paine‐Saunders et al., 2002), it is possible that BMP2 also serves as the binding intermediary between noggin and HSPG. We have prior demonstrated that noggin can bind and inhibit BMP2 prebound to ECM (Phillippi et al., 2008) suggesting that the complex formation could potentially be initiated via BMP2 and not noggin. Lastly, noggin inhibition studies performed during this study further suggest that the release of recycled ‘free’ BMP2 is unlikely to be involved in eBMP2‐EV bioactivity, whereby the recycled ‘free’ BMP2 binds to cell surface receptors, because the extracellular noggin remains available to sequester any non‐EV encapsulated, ‘free’ BMP2.

Based on current endocrine dogma, canonical and non‐canonical cell signalling via extracellular growth factors, including BMP2, is initiated upon GF binding to responsive cell surface receptors either directly or through an intermediary molecule. However, the role of internalization of these protein‐based ligands in signalling is less straightforward. Internalization of GF‐receptors occurs via endocytosis, which can result in subsequent signal downregulation, signal maintenance or generation of additional signalling (Dobrowolski & De Robertis, 2012). Signalling via eBMP2‐EV appears to bypass the cell surface BMP2 receptors. Several lines of evidence from the current study support this immediate bypassing of BMP2 surface receptors. First, EV luminal BMP2 retention confirmed not only acid prerinsing but also following trypsin EV surface treatment. Second, BMP2 was demonstrated to be 100% retained in eBMP2‐EVs for a minimum of 24 h. Lastly, eBMP2‐EVs were not subject to noggin inhibition, even under extended culture conditions. eBMP2‐EV binding and internalization are therefore likely dependent upon EV internalization mechanisms which include binding to cell surface HSPG (Cerezo‐Magaña et al., 2020; Gonda et al., 2019). Post‐HSPG binding, eBMP2‐EVs can then be subject to internalization via CCPs and CAVs dependent endocytosis into endosomes. These internalized eBMP2‐EVs in the early endosome still retain the BMP2 intralumenally, therefore even if BMP2 cell surface receptors are co‐internalized, BMP2‐to‐BMP2 receptor interaction remains unlikely to occur.

Similarly, any endocytosed noggin remains in the lumen of the endosome, also unavailable to interact with EV luminal BMP2. However, upon acidification of the endosome, the EV membrane fuses with the endosome membrane (Joshi et al., 2020), enabling the release of EV luminal BMP2 directly into the cytosol, while the BMP2 receptors and noggin remain in the lumen of the endosome. This is supported by our internalization experiments suggesting eBMP2‐EVs appears to traffic from cell surface to the cytoplasm. Co‐fluorescent staining of both EV and BMP2 suggest that internalized eBMP2‐EVs remain intact but upon trafficking perinuclearly within endosomes are subject to the release of BMP2 from eBMP2‐EVs. We hypothesize that this cytosolic BMP2 likely forms complexes with BMP Type I and II receptors within the cytosol. This results in the initiation of both canonical SMAD‐dependent and independent BMP2 signalling pathways, similar to cell surface BMP2 binding to its cell surface receptors, which ultimately results in similar osteoblastic gene transcription. Our data showed that eBMP2‐EVs induced time‐dependent transcription of *Msx2, Runx2, Dlx3, Dlx5, Alpl* and *Osx* in C2C12 and MC3T3 cells (Figure 6). *Runx2* is a downstream target of BMP signalling and is an essential transcription factor for the control of osteoblast differentiation. Previous reports have established that BMP2‐induced *Runx2* is mediated by *Msx2*, *Dlx3* and *Dlx5* (Hassan et al., 2006). While others have shown that BMP2 regulates *Osx* through *Msx2* and *Runx* (Celil & Campbell, 2005; Matsubara et al., 2008).

**FIGURE 8:**
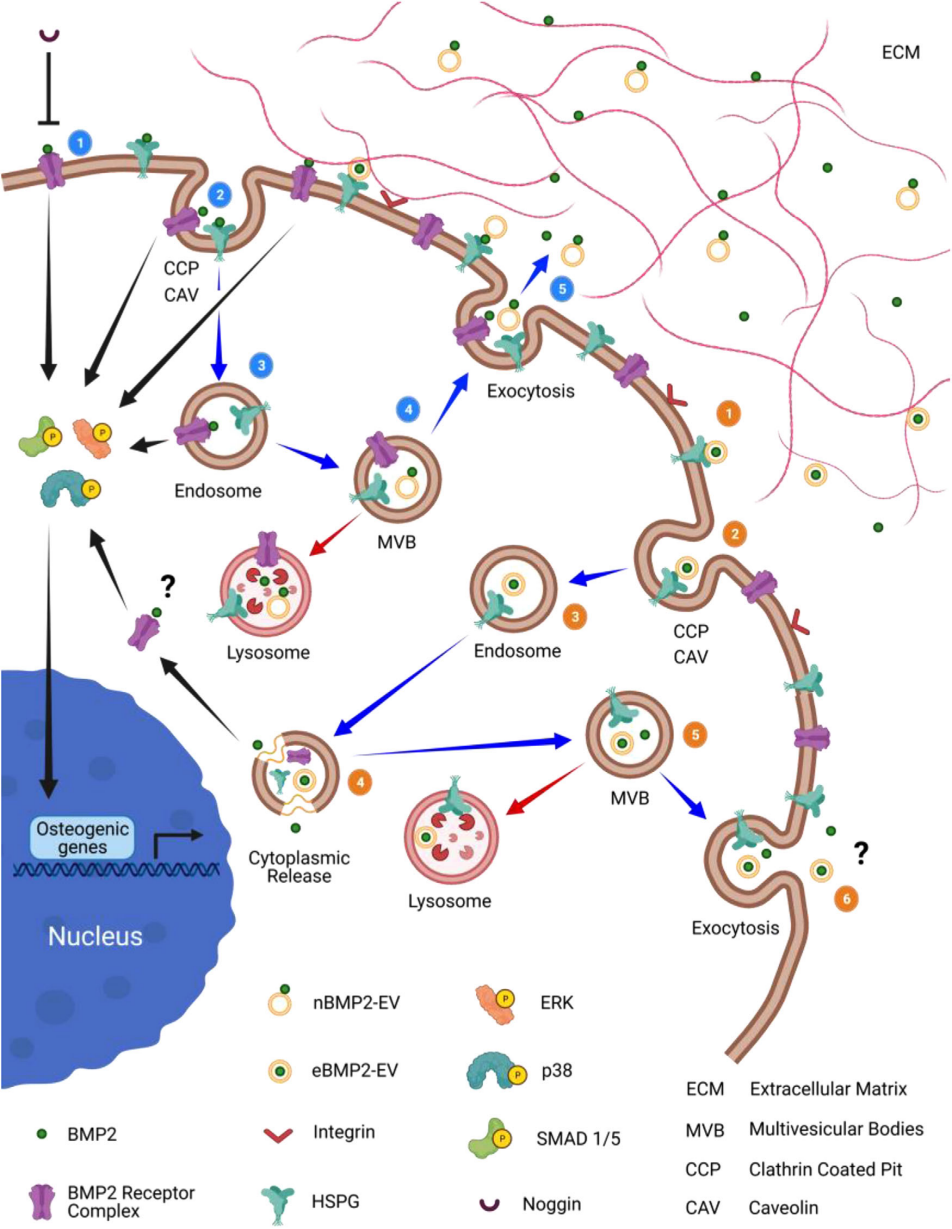
Schematic showing proposed mechanism for BMP2 signalling, cell trafficking, and recycling of BMP2, BMP2 receptors and HSPG, including the formation of nBMP2‐EVs. And, the differential signalling via eBMP2‐EVs and its role in BMP2 signalling bypassing both cell surface BMP2 receptors and noggin. Numbers within blue circles reflect the order of trafficking events for BMP2 signalling, beginning with receptor binding (1) through to recycling (5). nBMP2‐EV is subject to signalling via the BMP2 pathway. Numbers within orange circles reflect the order of trafficking events for eBMP2‐EV signalling, beginning with receptor binding (1) through to recycling (6). Arrows provide additional directional cues. Blue arrows indicate trafficking pathway, black arrows indicate signalling pathway and red arrows indicate delivery to lysosome degradation pathway. Created with BioRender.com

Our proposed model (Figure 8) incorporates the current literature within the context of the current study toward explaining a more complicated BMP2 signalling process involving not only BMP2 cell recycling but also EVs.

**FIGURE 9:**
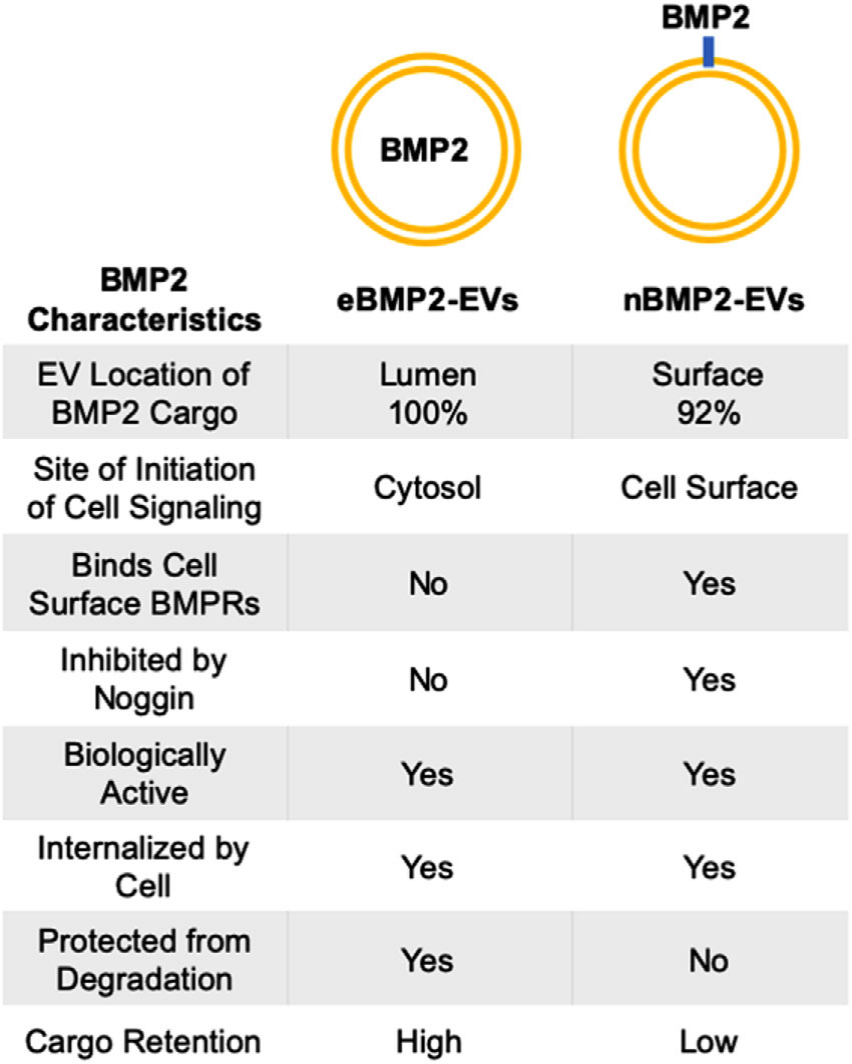
Table contrasting eBMP2‐EVs to nBMP2‐EVs

We found significant differences between nBMP2‐EVs and eBMP2‐EVs depending on where the BMP2 cargo was located. Figure 9 directly contrasts BMP2 as EV signalling cargo, comparing eBMP2‐EVs to nBMP2‐EVs. The characteristics reported here for nBMP2‐EVs are similar to those reported for BMP2/4‐EVs in the regulation of embryonic development in zebra fish (Draebing et al., 2018). However, considering clinical therapeutic translation, the characteristics of eBMP2‐EVs offer distinct advantages to nBMP2‐EVs, with the potential to greatly reduce the effective dosage of delivered BMP2 without being subject to extracellular degradation or inhibition by BMP2 inhibitors.

We used our inkjet‐based bioprinting technology (Campbell & Weiss, 2007) to create eBMP2‐EV solid‐phase microenvironments using collagen type I‐based substrates, tested them in vitro and in vivo, toward demonstrating that beyond the immobilization of bioactive ‘free’ BMP2 within the ECM, EVs can also enable immobilized reservoirs of BMP2 within the ECM. We reasoned that since GFs are found in the ECM in picogram to nanogram quantities (Taipale & Keski‐Oja, 1997), we would consider a similar concentration range for GFs as EV cargo in the cell microenvironment. Therefore, for in vitro studies we printed 2.4 pg to 9.6 pg (10 – 40 OPs) of total EV protein per pattern (1.25 × 1.75 mm), whereas for in vivo studies we printed approximately 280 ng EVs containing 5 ng BMP2 per construct (4.5 mm diameter disc). Although we printed highly defined microenvironments for in vitro studies, we observed that the ALP response was not strictly confined to this printed region, where cells around the perimeter of the printed pattern also staining positive for ALP expression. One potential explanation for the ‘spillover‐effect’ could be that the cells on‐ and off‐pattern communicate in a paracrine manner affecting each other's fate, and/or it could also represent the on/off EV ECM binding kinetics that resulted in upregulation of ALP expression in vitro (Figure 7b‐c), while nanogram quantities of eBMP2EVs resulted in HO in vivo (Figure 7e‐f). These results are in agreement with our previous reports on solid‐phase presentation of GFs, where we demonstrated regulation of stem cell fates using picogram quantities of immobilized GFs in vitro (Ker et al., 2011; Miller et al., 2009; Phillippi et al., 2008) and induction of osteogenesis in vivo using nanogram quantities of solid‐phase BMP2 (Brooker et al., 2019; Cooper et al., 2010a). The purpose of the current in vivo experiment was to demonstrate that bioprinted eBMP2‐EVs retained bioactivity as reflected by their ability to induce bone formation in vivo in an ectopic model (Scott et al., 2012). Experimental induction of ectopic bone has long been utilized in bone tissue engineering applications, including muscle pocket, subcutaneous and kidney capsule models and it remains the ‘gold standard’ in vivo bioassay to assess BMP2 bone inductive capacity (Scott et al., 2012). Rodent models, especially mice, are most commonly utilized for ectopic bone studies (Mcgovern et al., 2018). Therefore, we utilized a murine muscle pocket model as a first pre‐clinical demonstration of BMP2 osteoinductivity when delivered as eBMP2.

## 5 CONCLUSION

EVs were shown to be effective carriers for delivery of exogenous EV intralumenal BMP2. eBMP2‐EVs were internalized by cells and were biologically active, inducing osteogenic differentiation in vitro. Furthermore, cell trafficking and noggin inhibition studies collectively suggest that EV encapsulated BMP2 signalling is not initiated via binding to its cell surface receptor but results following the direct intracellular transfer of intact eBMP2‐EVs. Cell trafficking experiments with ‘free’ BMP2 suggest that nBMP2‐EVs can form by innate biological processes associated with BMP2 recycling which may play a role in innate BMP2 cell signalling. However, in contrast to eBMP2‐EVs, nBMP2‐EVs had BMP2 bound on the EV membrane surface which was susceptible to proteolysis and heparin, as well as exhibiting cell surface receptor binding and inhibition by extracellular noggin. It is interesting to speculate whether other GFs, engineered similar to eBMP2‐EVs behave similarly or whether this is unique to BMP2? Signalling mechanisms of cytosolic BMPR signalling via EV‐endosome fusion release of EV luminal BMP2 remains to be resolved.

The capability of using well‐defined bioprinted solid‐phase EV‐based microenvironments will facilitate such studies. Bioprinted solid‐phase eBMP2‐EV microenvironments resulted in spatially controlled osteogenic differentiation in vitro using picogram‐level dosages and induction of osteogenesis in vivo using nanogram‐level dosages. Localized delivery of solid‐phase GF‐EVs via bioprinted constructs could potentially reduce the therapeutic dosages of GFs required for desired therapeutic outcomes. The ability of eEVs to facilitate potent growth factor delivery beyond that of nEV surface delivery makes them an exciting class of delivery materials with potential uses in a myriad of therapeutic applications, including tissue engineering and regenerative medicine.

## CONFLICT OF INTEREST

The authors report no conflict of interest.

## Supporting information

Supporting Information

Supporting Information

Supporting Information

## Data Availability

Raw data is available from the corresponding author upon reasonable request. Raw/processed data required to reproduce these findings cannot be shared at this time as the data also forms part of an ongoing study.
