# Supplementary material for: Cell trafficking and regulation of osteoblastogenesis by extracellular vesicle associated bone morphogenetic protein 2
Source: J Extracell Vesicles. 2021 Oct 20;10(12):e12155. doi: 10.1002/jev2.12155 (PMC8528095; doi:10.1002/jev2.12155)
Supplement: Supplementary file 1 — Supporting Information [file JEV2-10-e12155-s002.docx]

**SUPPORTING INFORMATION**

**Cell Trafficking and Regulation of Osteoblastogenesis by Extracellular Vesicle Associated Bone Morphogenetic Protein 2**

*Saigopalakrishna S. Yerneni, Juraj Adamik, Lee E. Weiss and Phil G. Campbell*

**1. Additional Materials and Methods**

**1.1. EV nomenclature**

Over the past 50 years investigators have referred to extracellular vesicles using dozens of different classification criteria for naming different types of vesicles released from prokaryotic and eukaryotic cells, such as matrix vesicles, exosomes, nanovesicles, microvesicles and microparticles, just to name a few. However, since the myriad of identified types were based on different criteria and outdated isolation and detection techniques, the single term ‘EV’ was introduced by International Society on Extracellular Vesicles (ISEV) to avoid confusion and to improve the exchange of information between investigators and societies.

**1.2. Cell culture**

Mouse J774A.1 cells (ATTC^®^ TIB-67™, Manassas) were grown and maintained in Roswell Park Memorial Institute medium (RPMI, Gibco, Gaithersburg, MD) supplemented with 10% heat-inactivated fetal bovine serum (HI-FBS; Invitrogen, Carlsbad, CA) and 1% penicillin-streptomycin (PS; Invitrogen, Carlsbad, CA). Mouse C2C12 cells (ATCC^®^ CRL-1772^™^, Manassas, VA) were grown in Dulbecco’s Modified Eagle’s Media (DMEM; Invitrogen, Carlsbad, CA) containing 10% HI-FBS and 1% penicillin-streptomycin. MC3T3-E1 subclone 4 cells (ATCC^®^ CRL-2593^™^, Manassas, VA) were grown in ascorbic acid-free α-minimum essential media (αMEM, Gibco, Gaithersburg, MD) media supplemented with 10% HI-FBS and 1% PS. In all cell culture experiments, EV-depleted FBS obtained by centrifugation at 100,000xg for 2 hr was utilized. Conditioned media was collected every 72 hr and stored at -80 °C if not used immediately for EV isolation.

**1.3. Isolation of EVs**

EVs from conditioned media were isolated by size exclusion chromatography (SEC) using previously described protocol^1^. Briefly, conditioned media was centrifuged at 2,000*×*g for 10 min at 4°C and then at 10,000*×*g for 30 min at 4 °C. Supernatant was passed through a 0.22 µm-pore Millipore filter and EVs isolated by mini-SEC using 1.5 cm×12 cm mini-columns (Bio-Rad, Hercules, CA, USA; Econo-Pac columns) packed with 10 ml of Sepharose 2B (Sigma-Aldrich, St. Louis, MO, USA) equilibrated with phosphate-buffered saline (PBS). Supernatant (1.0 ml) was loaded onto the column and five 1 ml fractions corresponding to the void volume peak were collected by running PBS over the column. Fraction 4 was used for subsequent experiments. Isolated EVs were either used immediately (within 24 hr) for subsequent experiments or stored at -80 °C for long term storage.

**1.4. Characterization of EVs**

EVs were characterized by quantifying the protein content, transmission electron microscopy (TEM), tunable resistive pulse sensing (TRPS) and western blotting (see section on immunoblotting) for EV (exosome) surface markers as described previously and according to the current MISEV2018 guidelines^2^. EV concentrations are reported in µg/ml EV protein. Detailed experimental procedures are included in the supplemental information.

**1.5. Tunable resistive pulse sensing (TRPS)**

TRPS system by qNano (Izon, Cambridge, MA, USA) was used to measure the size distribution and concentration of particles in isolated EV fractions as previously described^1^. 40 µl EV suspension or calibration particles included in the reagent kit (2:1, 114 nm, Izon) were placed in the Nanopore (NP100 # A28126, Izon). All samples were measured at 45.06 mm stretch at 0.64 V and 11 mbar pressure. Particles were detected in short pulses of the current (blockades). The calibration particles were measured directly before and after the experimental sample under identical conditions. The sizes and concentrations of particles were determined using software provided by Izon (version 3.2).

**1.6. Transmission Electron Microscopy**

TEM characterization was performed as previously described^1^. Briefly, isolated total EVs were fixed with 4% glutaraldehyde (Electron Microscopy Services, Hatfield, PA, USA) for 20 min at RT. A 10 μL droplet of glutaraldehyde- fixed EVs was placed on Formvar-coated 300 mesh copper grid (Electron Microscopy Services, Hatfield, PA). The sample was incubated for 1min followed by rinsing with distilled water for 1 min to ensure removal of PBS salts. Excess liquid was blotted-off with a Whatman filter. Post rinsing, 50 µl of Uranyl-acetate solution was put on the grid and allowed to remain for 1 min. Excess liquid was removed, and the grids were viewed on a Hitachi H-7100 transmission electron microscope (TEM, Hitachi High Technologies) operating at 100 keV. Digital images were collected using an AMT Advantage 10 CCD Camera System (Advanced Microscopy Techniques) and inspected using NIH ImageJ software.

**1.7. Flow cytometry tracking**

BMP2 was labelled with Alexa Fluor 647 using a Microscale Protein Labeling Kit (Thermo Fisher Scientific, Waltham, MA) as previously described^3^. PKH26-labeled EVs were loaded with Alexa Fluor 647-labeled BMP2. MC3T3 cells were treated with Alexa Fluor 647-BMP2, PKH26-EVs or eBMP2-EVs for 4 hr and then analyzed for green and red fluorescence, with and without acid rinsing (See section 5.8.2 below.). Flow cytometric analysis was performed on an Accuri C6 flow cytometer (BD Biosciences, San Jose, CA) connected to an Intellicyt HyperCyt autosampler (IntelliCyt Corp., Albuquerque, NM) using green (488 nm) and red (649 nm) channels. Data were analyzed using FlowJo^®^ software (Flowjo LLC, Ashland, Oregon).

**1.8. Confocal microscopy**

PKH26-labeled EVs were loaded with Alexa Fluor 647-labeled BMP2 and incubated with MC3T3 cells for designated time points. To remove plasma membrane-bound EVs, cells were treated with stripping buffer (500 µM NaCl and 0.5 % acetic acid in deionized water, pH: 3) for 45 seconds followed by three washes with PBS. Cells were fixed with 3.33% freshly prepared paraformaldehyde (Electron Microscopy Services, Hatfield, PA) for 20 min at room temperature (RT). Excess fixative was quenched by adding an equal volume of 1% (w/w) BSA in PBS for 5 min followed by three washes with PBS. Fixed cells were permeabilized with 0.1% Triton X-100 in PBS for 1 min. To visualize F-actin and nuclei, cells were stained with Alexa Fluor 488-Phallodin (5:200 in PBS; Thermo Fisher Scientific, Waltham, MA) and Hoechst 33342 (1:1000 in PBS; Thermo Fisher Scientific, Waltham, MA), respectively. Imaging was performed using a Carl Zeiss LSM 880 confocal microscope with fixed settings across all of the experimental time points, and the images were analyzed using ZEN Black software (Carl Zeiss Microscopy, Thornwood, NY).

**1.9. Bioprinting**

Bioprinting of eBMP2-EVs was accomplished using our previously established inkjet-based bioprinting system^1^. A piezoelectric drop-on-demand printhead with a diamond-like carbon-coated 60 µm diameter nozzle (MicroFab Technologies, Inc., Plano, TX) was used for these experiments. A dilute EV bioink consisted of 100 µg/ml eBMP2-EVs (0.018ng BMP2/ng EV) in PBS with 10% glycerol. All the inks were degassed for 10 min prior to printing. The concentration of deposited BMP-EVs at individual locations on printing substrates or scaffolds was modulated using an overprinting strategy whereby the concentration increases with the number of overprints (OPs) ^1^. To validate the printing process, defined patterns of fluorescently labeled eBMP2-EVs (Alexa Fluor 647-labeled BMP2 and PKH26-labeled EVs) were printed on collagen type I coated coverslips (Neuovitro, Vancouver, WA) as 1.25 x 1.75 mm patterns arranged in 2 x 2 dose-modulated arrays of 5, 10, 15, and 20 OPs, with adjacent drop spacings of 80 µm.

For *in vitro* biological response experiments, collagen type I coated coverslips were printed as described above but with 10, 20, 30, and 40 OPs. After printing, the coverslips were rinsed with PBS overnight at 4 °C before seeding with C2C12 cells. After 3 days under cell culture conditions, coverslips were stained for ALP activity. For the solid-phase *in vivo* studies, 50 OPs of eBMP2-EVs or sonicated EVs without BMP2 controls were uniformly printed on 200 µm thick, 4.5 mm diameter discs of ADM (DermaMatrix^TM^, MTF Foundation, Edison, NJ), where the bioinks were absorbed into the discs. We have used this thin, collagen-rich material in prior feasibility bioprinting studies using small animal models in order to accommodate implantation into defects^4^.

**1.10. Micro-computed tomography (µCT) analysis**

Bone formation was analyzed using a VivaCT 40 (SCANCO Medical AG, Bassersdorf, Switzerland) µCT system. The femur and tibia, with surrounding soft tissue, were imaged in 70% ethyl alcohol inside a holder provided by the manufacturer and stabilized with parafilm. Imaging was performed using a 30 µm voxel size and the following conditions: 55 kVp/145 µA, FOV/Diameter of 30 mm, and single frame-averaging. 3D reconstruction of the scanned volumes with raw files was done automatically by the system’s operating software. The SCANCO µCT 3D morphometry and analysis software, which operates in an open VMS environment, was used for analysis. Region of interests (ROIs) were drawn by tracing the borders of the heterotopic bone formed around the thigh region where the ADM scaffolds were implanted, and a global threshold of 158.4 mg HA/ccm was applied for the heterotypic bone/background segmentation. The thresholding value represented the peak characteristic of bone tissue in the gray value distribution histogram as previously described^5^. The ROI regions were assessed for bone volume (BV).

**1.11. Decalcification and histology**

After micro-CT analysis, legs were placed in nylon mesh bags (Electron Microscopy Sciences, Hatfield, PA) and subjected to Cal-Rite™ solution (Richard-Allan Scientific™, San Diego, CA) at RT for two weeks with constant agitation. The thigh region consisting of the implant was sectioned and embedded in paraffin blocks for histological analysis. The paraffin embedded specimens were sectioned at a thickness of 5 µm and stained with hematoxylin and eosin and Masson’s trichrome stain.

**2. Supplemental Figures**


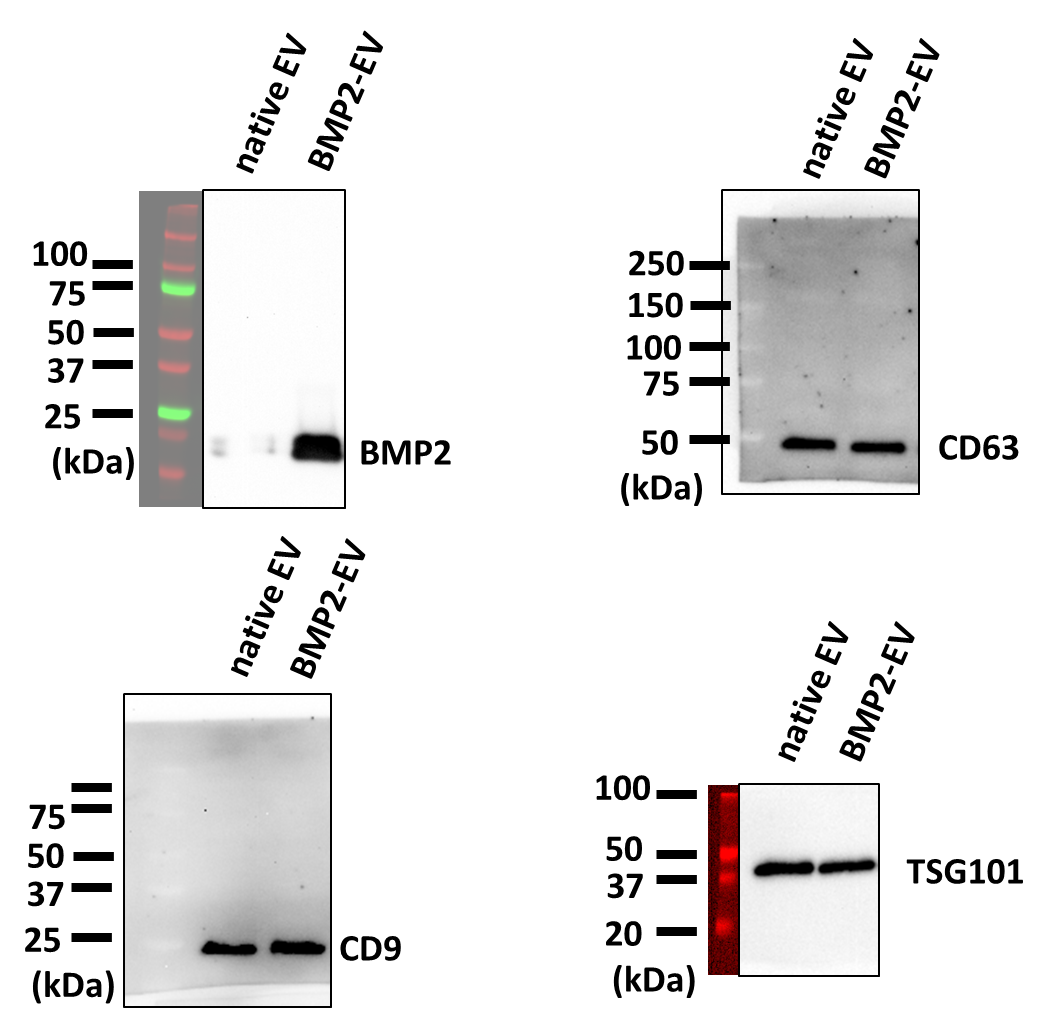


**e**

**e**

**e**

**e**

**A**

**B**

**D**

**C**

**Supplementary Figure S1**. Full blot images of WB data shown in Fig. 1(C). Blots include (**A**) BMP2, (**B**) CD63, (**C**) CD9 and (**D**) TSG101.


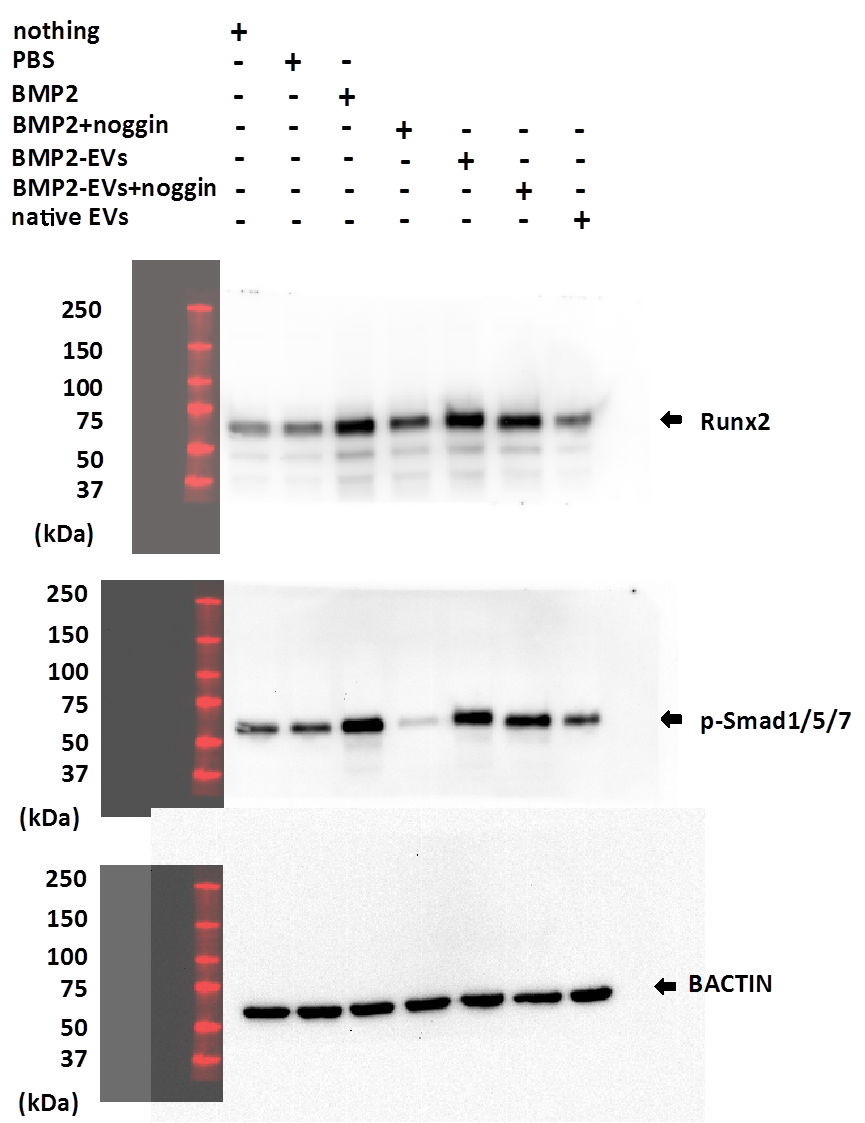


**e**

**e**

**Supplementary Figure S2**. Full blot image of WB data shown in Fig. 2(C) for C2C12 cells.


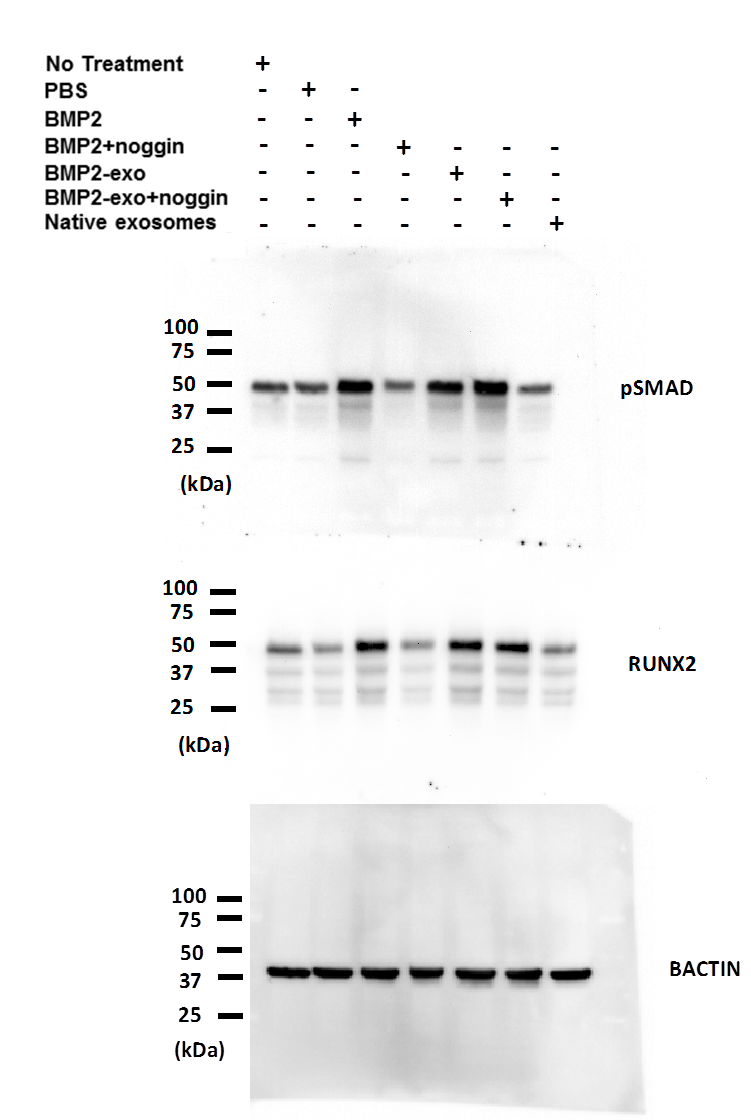

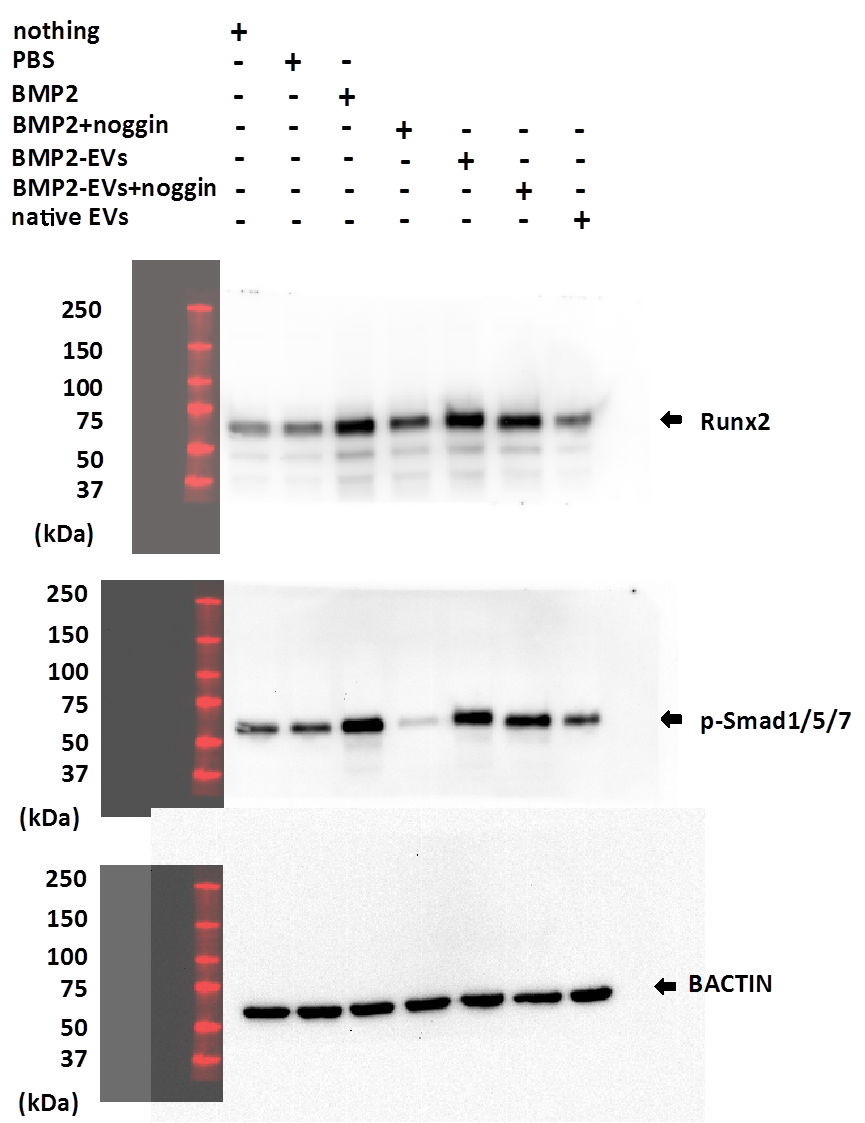


**e**

**e**

**Supplementary Figure S3**. Full blot image of WB data shown in Fig. 2(C) for MC3T3 cells.


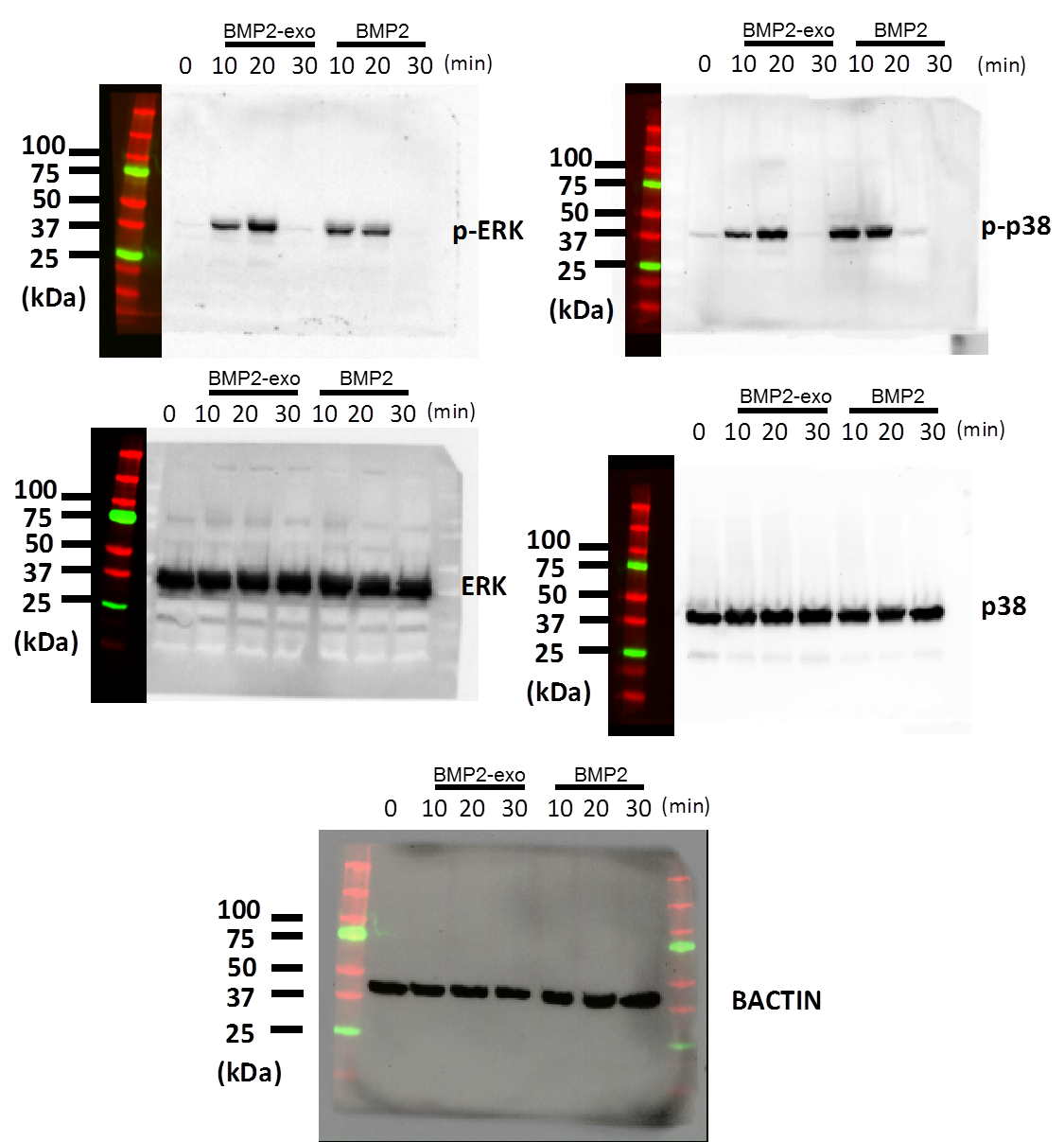


**eBMP2-EVs BMP2**

**eBMP2-EVs BMP2**

**eBMP2-EVs BMP2**

**eBMP2-EVs BMP2**

**eBMP2-EVs BMP2**

**Supplementary Figure S4**. Full blot image of WB data shown in Fig. 2(B).


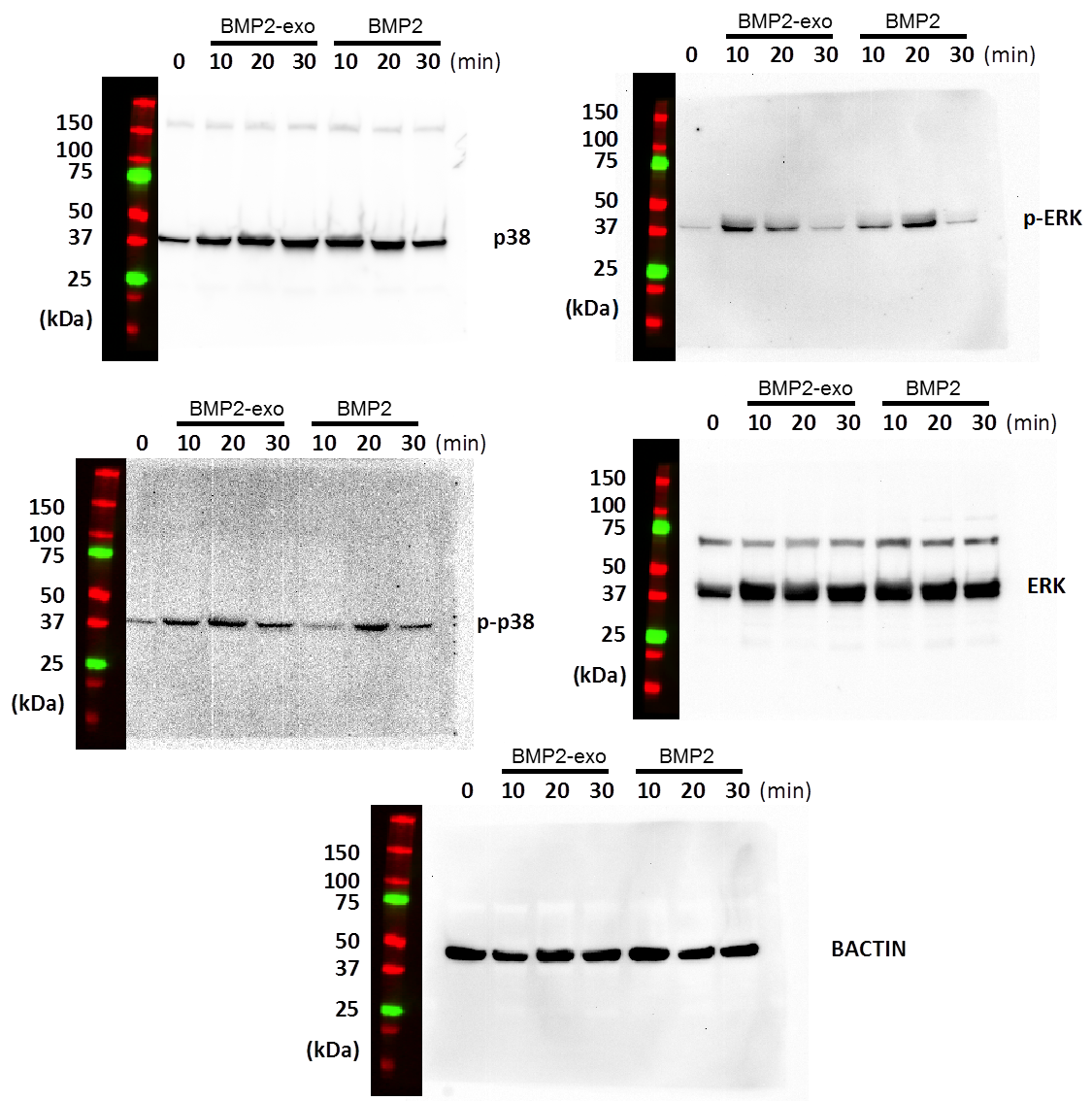


**eBMP2-EVs BMP2**

**eBMP2-EVs BMP2**

**eBMP2-EVs BMP2**

**eBMP2-EVs BMP2**

**eBMP2-EVs BMP2**

**Supplementary Figure S5**. Full blot image of WB data shown in Fig. 2(C).


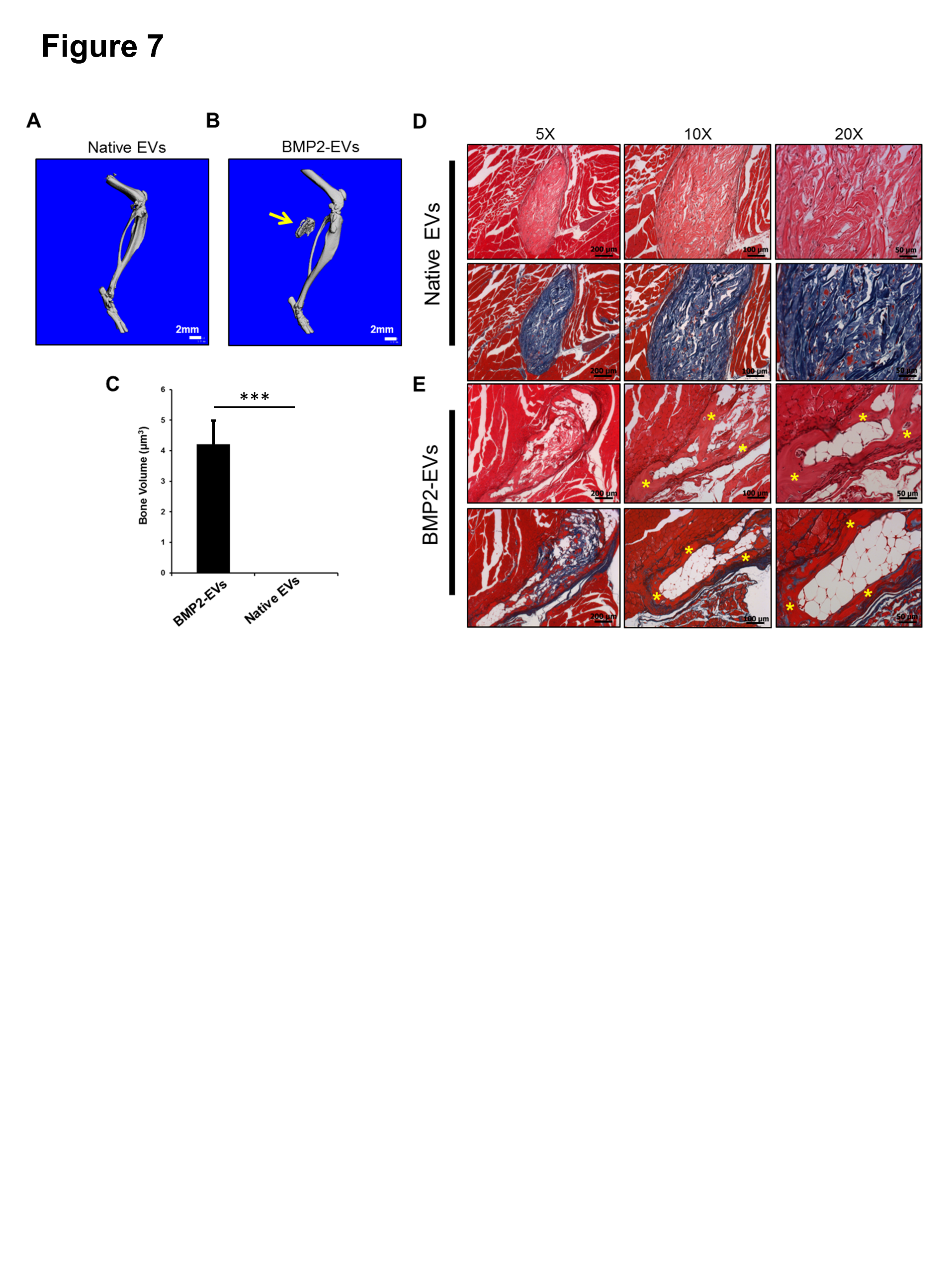


**e**

**Supplementary Figure S6**. µCT quantification of heterotopic ossification with eBMP2-EVs and native EV bioprinted constructs in murine muscle pocket model shown in Figure 7E, ***=ρ≤0.001.

| **Gene** | **Forward primer (5′->3′)** | **Reverse primer (5′->3′)** |
| --- | --- | --- |
| ***18srRNA*** | GAGCGACCAAAGGAACCATA | CGCTTCCTTACCTGGTTGAT |
| ***Runx2*** | CCTCTGACTTCTGCCTCTGG | ATGAAATGCTTGGGAACTGC |
| ***Ocn*** | TAGTGAACAGACTCCGGCGCTA | TGTAGGCGGTCTTCAAGCCAT |
| ***Bsp*** | AAGAAGAGGAAGAGGAAGAAAATGA | GCTTCTTCTCCGTTGTCTCC |
| ***Osx*** | AGAGGTTCACTCGCTCTGACGA | TTGCTCAAGTGGTCGCTTCTG |
| ***Alpl*** | CACGGCCATCCTATATGGTAA | CTGGGCCTGGTAGTTGTT |
| ***Dlx3*** | GTACCGGGAGCAGCCTTT | CTTCCGGCTCCTCTTTCA |
| ***Dlx5*** | GCCCCTACCACCAGTACG | TCACCATCCTCACCTCTG |
| ***Msx2*** | ATACAGGAGCCCGGCAGATA | CGGTTGGTCTTGTGTTTCCT |

**Supplementary Table S1**. qPCR Primers for mRNA analysis.

**
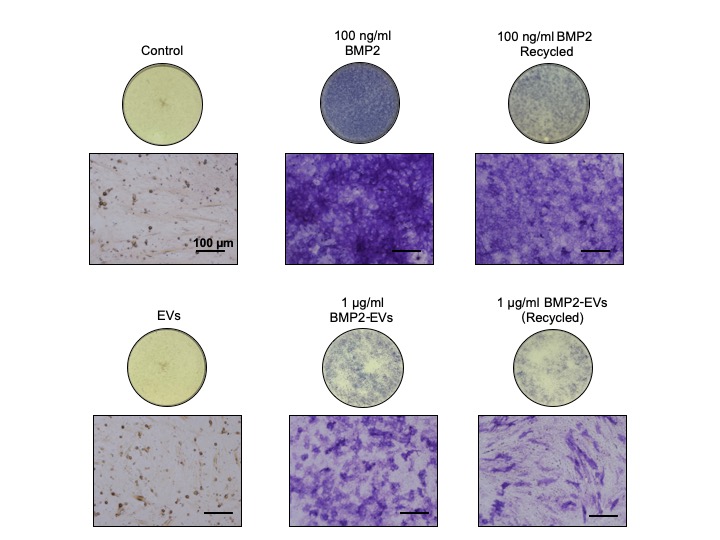
**

**n**

**1 µg/ml nBMP2-EVs**

**Recycled**

**Supplementary Figure S7**. Representative images of ALP staining for accessing the bioactivity of recycled “free” BMP2 and nBMP2-EVs.

**Supplementary Movie S1**. Confocal Z-stack 3D rendering of eBMP2-EVs internalized by C2C12 cells.

**Supplementary Movie S2**. Confocal Z-stack 3D rendering of eBMP2-EVs internalized by MC3T3 cells.

**3. References**

1. Yerneni, S.S., L., W.T., Weiss, L.E. & Campbell, P.G. Bioprinting exosome-like extracellular vesicle microenvironments. *Bioprinting*, e00041 (2019).

2. Thery, C.*, et al.* Minimal information for studies of extracellular vesicles 2018 (MISEV2018): a position statement of the International Society for Extracellular Vesicles and update of the MISEV2014 guidelines. *J Extracell Vesicles* **7**, 1535750 (2018).

3. Alborzinia, H.*, et al.* Quantitative kinetics analysis of BMP2 uptake into cells and its modulation by BMP antagonists. *J Cell Sci* **126**, 117-127 (2013).

4. Cooper, G.M.*, et al.* Inkjet-based biopatterning of bone morphogenetic protein-2 to spatially control calvarial bone formation. *Tissue Eng Part A* **16**, 1749-1759 (2010).

5. Zaky, S.H.*, et al.* Poly (glycerol sebacate) elastomer supports bone regeneration by its mechanical properties being closer to osteoid tissue rather than to mature bone. *Acta Biomater* **54**, 95-106 (2017).
